# Data mining historical Chinese medical recipe collections and nuclear receptor profiling identify plant fractions that modulate glucocorticoid receptor activity

**DOI:** 10.3389/fphar.2025.1681729

**Published:** 2026-01-07

**Authors:** Joachim Prackwieser, René Houtman, Tim Kievits, Diana Melchers, Haifeng Guan, Georg Seifert, Frank Konietschke, Kai Lamottke, Paul U. Unschuld, Nalini Kirk

**Affiliations:** 1 Institute for Chinese Life Sciences, Charité - Universitätsmedizin Berlin, Corporate Member of Freie Universität Berlin and Humboldt Universität zu Berlin, Berlin, Germany; 2 Precision Medicine Laboratory, Oss, Netherlands; 3 Bicoll Biotechnology (Shanghai) Co. Ltd., Shanghai, China; 4 Charité Competence Center for Traditional and Integrative Medicine, Charité - Universitätsmedizin Berlin, Corporate Member of Freie Universität Berlin and Humboldt Universität zu Berlin, Berlin, Germany; 5 Institute of Biometry and Clinical Epidemiology, Charité - Universitätsmedizin Berlin, Corporate Member of Freie Universität Berlin and Humboldt Universität zu Berlin, Berlin, Germany; 6 Bicoll GmbH, Planegg, Germany

**Keywords:** data-mining, glucocorticoid receptor, inflammation, natural products, psoriatic arthritis, Pareto front, rheumatoid arthritis, traditional Chinese medicine

## Abstract

**Objective:**

Glucocorticoids (GCs) play a prominent role in the management of chronic inflammatory diseases like rheumatoid arthritis and psoriatic arthritis, but their use is associated with various adverse effects. The therapeutic and adverse effects of GCs can be partly explained by modes of engagement with their target, the glucocorticoid receptor (GR). Identifying compounds that modulate GR through alternative mechanisms of action may provide a strategy to decouple therapeutic efficacy from side effects. Historical manuscripts on Chinese pharmacotherapy, which document the empirical use of natural products in treating inflammatory diseases, represent an underexplored source in drug discovery. These texts offer a historical library of plant materials and their metabolites, enabling strategic pre-selection of plant candidates for GR-targeted screening.

**Method:**

This study utilizes the Chinese Historical Healthcare Manuscripts Database, a newly compiled corpus comprising over 41,000 medical recipes from 227 historical Chinese manuscripts, to identify plant-based treatments for rheumatoid arthritis and psoriatic arthritis. Pareto front analysis, a multi-objective optimization method, was applied to 1,897 relevant recipes to identify plants that consistently ranked high across multiple metrics, suggesting their effectiveness in historical practice. The results were evaluated by comparison with modern Chinese *materia medica* dictionaries. Extracts from ten of these resulting plants underwent fractionation and were screened for GR modulation using Nuclear Receptor Activity Profiling (NAPing).

**Findings:**

Pareto front analysis identified 32 botanical drugs statistically associated with historical disease indications resembling rheumatoid arthritis, psoriatic arthritis, and psoriasis. Nineteen of these are explicitly described in Chinese *materia medica* dictionaries for the treatment of such diseases. None of the plant fractions tested by NAPing replicated classical GC-induced GR-coregulator binding, but three induced unique binding interactions, suggesting alternative GR modulation mechanisms.

**Conclusion:**

This study illustrates how combining data mining of historical pharmaceutical recipes with molecular screening can accelerate the discovery of new and possibly safer GR modulators. Such approaches may inform future translational strategies for treating chronic inflammatory diseases.

## 1 Introduction

Inflammation is the body’s natural response to injury, infection, or noxious stimuli. While acute inflammation is a short-term protective mechanism, chronic inflammation is a prolonged and maladaptive response, leading to tissue damage and impaired repair processes. Chronic inflammation plays a key role in many widespread diseases, not only in allergies and autoimmune disorders but also in atherosclerosis, cancer, and metabolic disorders (Hotamisligil, 2017; Lawler et al., 2021; Fernandes et al., 2024).

Rheumatoid arthritis (RA) and psoriatic arthritis (PsA) are two such immune-mediated diseases that primarily affect the joints and periarticular soft tissues. While they share certain clinical and immunological features, PsA is distinguished by its association with psoriatic skin or nail lesions. The global prevalence of RA ranges from 0.24% to 2.70%, and that of PsA up to 1%. These variations depend on genetic, socioeconomic, and demographic factors, as well as the quality of healthcare systems (Almutairi et al., 2021; Karmacharya et al., 2021).

Although synthetic disease-modifying antirheumatic drugs (DMARDs) and biologic therapies have brought great advances to treatment (Abbasi et al., 2019; Ogdie et al., 2020), glucocorticoids (GCs) remain a highly effective instrument to control inflammation. However, long-term or high-dose GC use is associated with adverse effects such as psoriasis flares and osteoporosis (Buttgereit, 2020; Vincken et al., 2022). Some of these beneficial and adverse effects, including GC resistance, can be explained by the function of the glucocorticoid receptor (GR) (Yang et al., 2021; Macfarlane et al., 2024). This has led to further research into the effects of GR modulators (van der Laan and Meijer, 2008) that maintain anti-inflammatory efficacy while minimizing side effects. In addition to synthetic drugs (Weatherley et al., 2018; van Laar et al., 2023), natural compounds have shown potential to modulate GR in the treatment of inflammatory diseases (Wang et al., 2023).

Chinese pharmacotherapy (CP) and other traditional medicines have used medicinal plants for millennia to treat inflammation. The anti-inflammatory effects of plant-derived secondary metabolites – particularly terpenoids and sterol-like compounds – have been described for a variety of botanical drugs (Gong et al., 2023; Wang et al., 2023; Jiang et al., 2024) used in contemporary traditional Chinese medicine (TCM). CP demonstrated its potential to inhibit inflammation and slow disease progression in RA and psoriasis, with certain botanical drugs enhancing efficacy and reducing side effects of biomedical treatments (Yu et al., 2017; Zhou et al., 2018; Wang et al., 2021; Li et al., 2022). Although these treatments have their origin in historical practice, systematic analysis of historical medical texts has rarely been conducted and offers a rich source for the discovery of new GR-targeting anti-inflammatory metabolites.

The example of artemisinin has proven the great potential of manual analysis of historical records as a source for drug discovery (Tu, 2016). More recently, computational approaches, driven by advances in text mining and deep learning, allow their systematic exploration (Connelly et al., 2020; Cheng et al., 2021; Zhang T. et al., 2022). These methods may help identify plants and bioactive metabolites whose therapeutic use was sustained over centuries for the treatment of specific diseases (Helmstädter and Staiger, 2014) such as RA and PsA. However, most CP studies that employ computational methods rely on datasets of contemporary TCM that reflect present-day prescription practices (Jo et al., 2022; Lai et al., 2022), and only a few have applied basic word-frequency analysis to printed historical texts (Xia et al., 2020; Zhang C. S. et al., 2022). Both types of studies lack access to the broad range of plants historically used in folk healing and tend to highlight well-known treatments while overlooking less familiar but potentially effective ones.

The historical Chinese medical recipes recorded in manuscripts of the ‘Unschuld Collection’ (Unschuld and Zheng, 2012; Crossasia.org, 2016) allow us to address such limitations. These hitherto mostly unexplored texts date to the late 19th and early 20th centuries but hold much older material transmitted over generations. Written down by their authors for private use, the recipe records reflect the actual historical practice of CP. In comparison to printed books, they largely avoid theoretical medical reasoning and document a much broader spectrum of medicinal plants, including locally used species absent from supra-regional commercial trade. The recently developed Chinese Historical Healthcare Manuscripts Database (CHHM-DB) makes these recipes amenable to data analysis, serving as an extensive library of botanical drugs from historical therapeutic practice. Nearly one-quarter of the recipes target musculoskeletal and skin disorders, making this dataset particularly valuable for studying RA and PsA.

A recent study found that recipe ingredients that repeatedly appeared in medieval texts yielded biologically active molecules of clinical relevance (Connelly et al., 2020). Similarly, the repeated appearance of the same or slightly varying recipes across different manuscripts in CHHM-DB suggests that effective recipe ingredients appear more frequently than non-effective ones in this large, systematic dataset. We hypothesize that not only frequently occurring botanical drugs but frequently *and* specifically used ones reflect historical preference and potential efficacy. While overall frequency may simply indicate common usage in Chinese medicine, specific usage is measured by the relative frequency for a given therapeutic indication compared to the whole dataset. These two attributes are inversely correlated as overall frequent recipe ingredients are often low in indication-specific frequency. Therefore, we apply Pareto front (PF) analysis, a multi-objective optimization method that balances both frequency and relative frequency.

This study pursues two main objectives: First, the recipes in CHHM-DB linked to indications resembling RA and PsA are investigated statistically through PF to identify botanical drugs that may have been historically preferred for their effectiveness in treating inflammation. Second, we investigate the potential of these botanical drugs to modulate GR, the target of drugs in GC-based treatments for inflammation.

GR belongs to the nuclear receptor (NR) family of proteins that control gene transcription for various biological processes. NR activity is regulated by ligands – endogenous substances or pharmaceutical drugs – that bind to specific sites in the NR ligand-binding pocket. Ligand binding to NRs results in the recruitment of a wide variety of coregulator proteins to DNA, where they modulate chromatin accessibility and transcription. The specific properties of a ligand strongly influence which coregulators are recruited, thereby shaping gene-specific transcriptional responses (Nettles and Greene, 2005; MacTavish et al., 2025). Moreover, different NR target genes require distinct combinations of coregulators, highlighting that context-dependent recruitment of coregulators is a key mechanism by which GR controls gene expression (Millard et al., 2013). This suggests that new GR-targeting drugs with improved therapeutic profiles and fewer side effects could be developed by selectively modulating coregulator recruitment to promote beneficial gene programs while minimizing adverse ones (van Moortel et al., 2024). Comparing GR-coregulator binding behavior induced by bioactive plant metabolites to those caused by GCs may help to identify candidates with reduced side effects (Xiang et al., 2018).

To screen for such potential alternative GR modulators, the plant materials identified by PF were fractionated and subjected to Nuclear Receptor Activity Profiling (NAPing), an *in vitro* screening technique that quantifies compound-mediated NR binding to a library of coregulator proteins (van Moortel et al., 2024). This approach provides a functional fingerprint of how plant metabolites modulate GR activity, offering higher-resolution insights than conventional gene reporter assays or crude plant extract testing.

We pursue a multidisciplinary reverse-translational methodology – moving from historical bedside to modern benchtop (Musyuni et al., 2023) – that integrates CP practice recorded in historical manuscripts with a molecular assay of GR engagement, offering a complementary path to conventional drug discovery.

## 2 Methods

### 2.1 Identifying Chinese search terms in CHHM-DB

The dataset of CP recipes used for statistical analysis in this study was derived from CHHM-DB. To build this database, 41,359 medical recipes from 227 handwritten recipe collections were extracted. These recipes are linked to 2,893 therapeutic indications and consist of altogether 3,638 recipe ingredients of botanical, animal, mineral, fungal, and other origins. The resulting data yields for each recipe record a title, a list of indications, a list of ingredients with standardized dosages and processing instructions, preparation and administration instructions, and an array of metadata.

Data mining CHHM-DB requires a list of therapeutic indications that serve as search terms to identify relevant recipes. When defining search terms, we had to keep in mind that retrospective biomedical diagnosis of historical disease entities remains a controversial issue (Cunningham, 2002; Mitchell, 2011) and can only be approximated based on clinical signs and observable symptoms.

In Chinese medicine, disease names may refer to specific disease entities, more general signs or symptoms, or complex concepts that describe the underlying pathophysiology. Moreover, historical medical texts, including the manuscripts in the Unschuld Collection, lack the systematic clinical descriptions of modern medicine (see Supplementary Data Sheet 1 for an exemplary original recipe entry). As a result, the therapeutic indications for the recipes in CHHM-DB only allow for an approximate biomedical identification, and defining search terms requires a balance between accuracy and the largest possible dataset.

**FIGURE 1:**
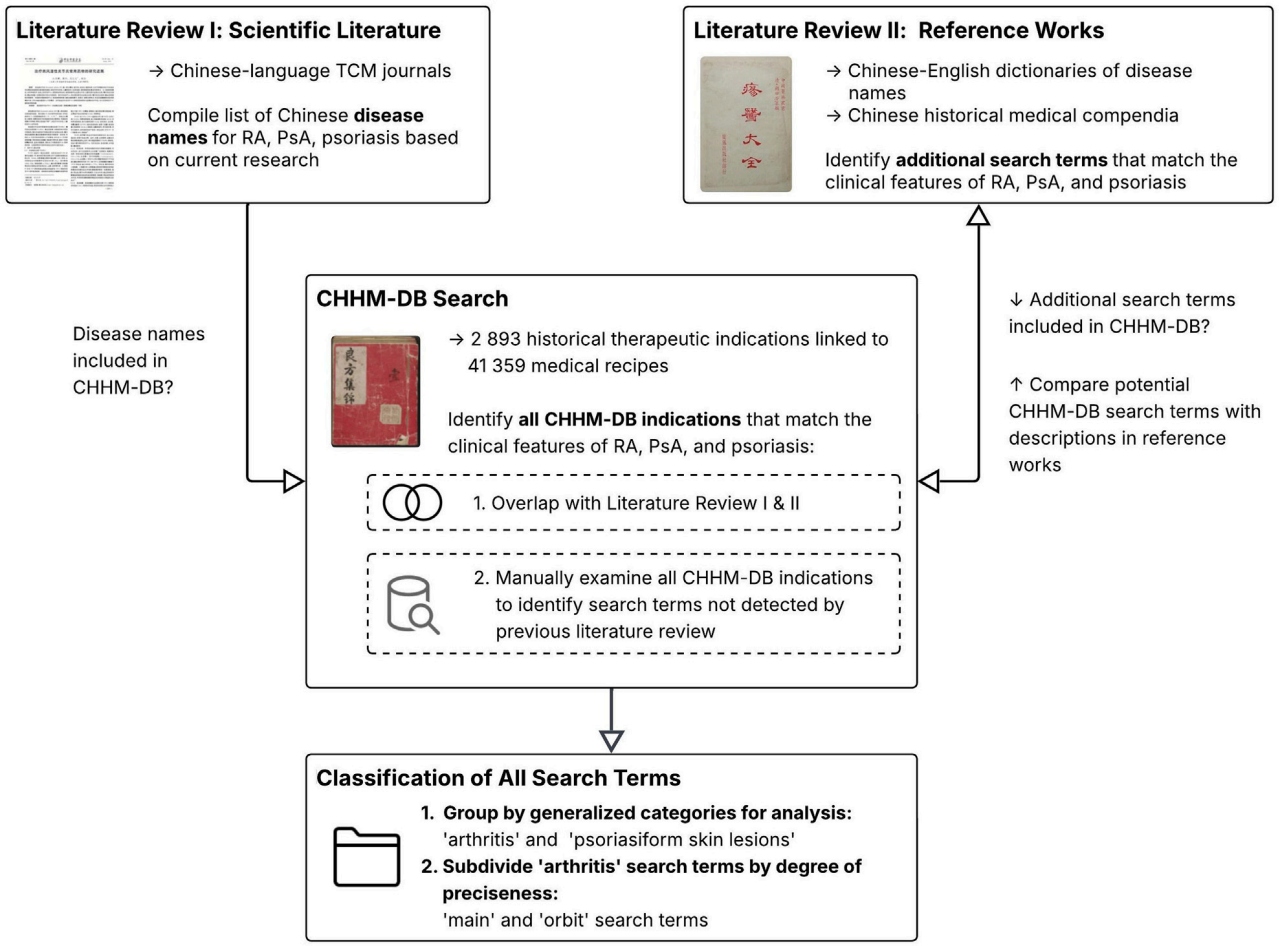
Flowchart depicting the process of identifying relevant search terms.

Joint inflammation in PsA is closely related to skin manifestations, and in conventional medicine similar anti-inflammatory drugs can be used for RA, PsA, or psoriasis. Therefore, search terms included not only Chinese designations for arthritis but also for psoriasis-like skin eruptions, which may point to recipes indicated for PsA and containing botanical drugs with anti-inflammatory effects on both joints and skin. To compile a list of search terms, the results of an extensive literature review were compared with the content of CHHM-DB (see Figure 1).

First, traditional Chinese disease names used for RA, PsA, and psoriasis in Chinese-language TCM journals were identified. In a second step, Chinese-English dictionaries of disease names (Lin, 2002; Zhang and Unschuld, 2015) and historical Chinese medical compendia (Sun, 1987; Gu, 1992; Ye, 1995) were browsed to identify additional search terms based on their resemblance to the clinical features of RA, PsA, or psoriasis. In the case of psoriasis, identification relied primarily on morphological descriptions of skin lesions found in historical texts. Finally, all indications listed in CHHM-DB were manually examined and, where a potential search term was identified, compared with its descriptions in the dictionaries and compendia.

Given the challenges of retrospective diagnosis outlined above, we group RA and PsA under the broader term “arthritis” and use the term “psoriasiform skin lesions” rather than “psoriasis” when referring to the historical source material.

### 2.2 Pareto front analysis

Analyzing a dataset defined by search terms covering two broad groups of illnesses required a multi-layered approach. Given the historicity of the data, we opted for an exploratory data analysis of the joint dataset containing all recipes indicated for arthritis and/or psoriasiform skin lesions (“All Search Terms”) first.

In a second step, to consider the varying preciseness of search terms, we differentiated the recipes for arthritis from those for psoriasiform skin lesions by defining four subsets from the joint dataset. These were defined as follows:

- “Arthritis Main and Orbit”: recipes linked to all arthritis search terms
- “Arthritis Main”: recipes linked to only precise arthritis search terms
- “Skin”: recipes linked to search terms resembling psoriatic skin lesions
- “Arthritis&Skin”: recipes linked to skin as well as arthritis search terms

By exploring these subsets, a better understanding could be gained of which plants were used specifically for arthritis or psoriasiform skin lesions, and their intersection, i.e., Arthritis&Skin. Non-plant recipe ingredients such as minerals and animal-derived substances, though present in CHHM-DB, were not included in the scope of this study.

To summarize the data, two summary metrics were generated for the joint dataset and each of the four subsets:

1. Frequency: number of occurrences of a botanical drug within the joint dataset or within a subset
2. Relative frequency: frequency of a botanical drug normalized to its total occurrences in the entire CHHM-DB (expressed as a percentage)

We hypothesized that effective recipes occur more frequently in CHHM-DB, making a botanical drug’s frequency a potential indicator of therapeutic relevance. However, since some drugs are common in CP irrespective of their specific efficacy, relative frequency was included as a complementary metric. The two variables showed an inverse relationship, indicating that multiple drugs would possess an optimal balance of both metrics.

To achieve this optimal balance between frequency and relative frequency, we employed PF analysis. A Pareto-optimal solution set consists of a family of optimal solutions that are non-dominating among each other but are superior to the rest of the solutions in the space of analysis. These Pareto-optimal solutions form the PF.

**FIGURE 2:**
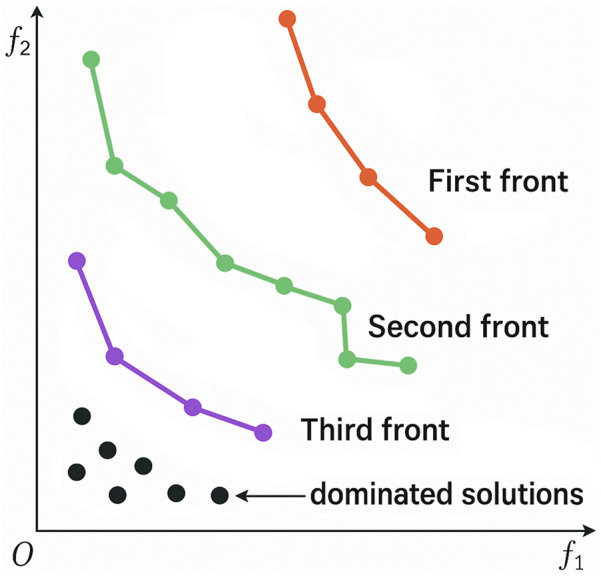
Schematic representation of Pareto front ranking. f_1_ denotes the frequency of botanical drugs in recipes linked to search terms, and f_2_ their relative frequency. The first front (rank1) is constituted by the set of Pareto-optimal solutions that are non-dominating among each other but superior to all remaining points. After its removal, all subsequent fronts are determined iteratively based on joint optimization of f_1_ and f_2_.

In a pool of solutions, PF rank determines solution fitness, i.e. the set of solutions can consist of multiple PFs. The family of optimal solutions that is superior to the rest of the solutions in the space of analysis constitutes rank 1. When this PF is removed, the solutions in the new front constitute rank 2. This ranking process continues until all solutions are classified (see Figure 2).

Three PFs were established for the joint dataset. Each subset was used to explore ranks 1*–*3 of plants that occur most frequently (frequency) as well as those specific to arthritis and skin illnesses (relative frequency).

The CHHM-DB derived dataset was analyzed by R statistical software (R Core Team, 2021). First, we used the *dplyr* (Wickham et al., 2021a) and *tidyr* (Wickham et al., 2021b) packages for data manipulation to format the data into the structure necessary to identify PFs. The *rPref* package (Roocks, 2016) was used to determine the PFs. Once PFs were established, we used the *ggplot2* (Wickham, 2016) package for visualization.

### 2.3 Evaluation of Pareto front results and comparison with contemporary TCM

To evaluate the botanical drugs revealed by PF analysis, they were compared with the respective monographs in Chinese *materia medica* (CMM) dictionaries (Xie, 1983; National Administration of TCM, 1999; Nanjing University of TCM, 2014) for relevance in traditional treatments of arthritis or psoriasiform skin lesions. Candidates from PF ranks 1*–*3 were assessed by two criteria: First, their specific usage in treating these conditions and, second, their level of toxicity.

Non-toxic plants whose CMM monographs predominantly list indications corresponding to the identified search terms and not a wide range of other indications were classified as “specific” and prioritized. Those used primarily for other indications but mentioned for arthritis and/or psoriasiform skin lesions were classified as “unspecific” and had lower priority. Toxic plants requiring pre-preparation to reduce toxicity were classified as “toxic” and excluded from screening, as this study focused on unprocessed botanical drugs.

For the identification of Chinese botanical drug names, we rely on authoritative CMM sources (Xie, 1983; National Administration of TCM, 1999; Nanjing University of TCM, 2014; National Pharmacopoeia Commission, 2020) but follow the updated binomial nomenclature in the Global Biodiversity Information Facility (GBIF.org, 2024). Where these modern species names differ substantially from synonyms still common in TCM literature, the latter are provided in square brackets. If more than one species was listed for a Chinese drug name in CMM, the species selected for screening was the one most commonly listed and widely used, which is also the one most frequently examined in pharmacological research. This ensured consistency with traditional usage and comparability with existing scientific literature.

To assess how well-known the botanical drugs identified from historical CP still are in contemporary TCM, we compared the PF results with entries in the 2020 edition of the official *Pharmacopoeia of the People’s Republic of China* (hereafter *Chinese Pharmacopoeia*) (National Pharmacopoeia Commission, 2020) and with the number of relevant publications indexed in PubMed. Literature searches were performed using combined queries that included the binomial name(s) of each plant (e.g., “Genus species” [Title/Abstract]) together with disease-specific or general inflammation terms. Searches were conducted for RA (“rheumatoid arthritis”), PsA (“psoriatic arthritis”), and psoriasis (“psoriasis”), and for inflammation more broadly (“inflammation” [Title/Abstract] OR “anti-inflammatory” [Title/Abstract]). If more than one botanical species was listed under a single Chinese drug name, all relevant species names were included in the query using the Boolean operator OR. Searches were conducted in September 2024 and updated after review in October 2025.

### 2.4 Preparation of plant fractions

Plant extraction and fractionation underwent the workflow routinely applied in previous studies (Helleboid et al., 2014; Laszlo et al., 2022). Powdered botanical drug material (0.5 g) was weighed into a 50 mL centrifuge tube, supplemented with 15 mL of extraction solvents of different polarity (drug-to-solvent ratio 1:30 w/v), and extracted in an ultrasonic bath for 20 min at room temperature. Suspensions were centrifuged at 10,000 rpm for 5 min, the supernatants filtered through 0.22 µm syringe membrane filters, and the filtrate was blow-dried with argon at room temperature. A nonpolar crude extract (obtained with dichloromethane [DCM] as solvent) was prepared from all selected plants, and, in addition, a more polar crude extract (obtained with EtOH as solvent) was prepared for one plant *Clematis chinensis* Osbeck [Ranunculaceae].

Each extract was then subjected to reverse phase separation using Bifrac N™ technology (Bicoll GmbH, Munich, Germany). 96 fractions were collected and subsequently freeze-dried to determine dry weight. The freeze-dried material of each fraction was re-dissolved to allow for aliquoting of 0.2 mg in a well of a 96-well plate. The plates, each now containing up to 96 fractions per extract, were again freeze-dried and stored until use.

### 2.5 Glucocorticoid receptor NAPing

Freeze-dried plant fractions were dissolved in 50 µL DMSO and incubated for 30 min at room temperature on an orbital shaker. Prepared solutions were analysed using GR NAPing as described previously (van Moortel et al., 2024). In brief, the technique measures *in vitro* how compounds influence the binding of GR to a library of 101 immobilized peptides that represent its natural interaction sites on coregulator proteins (motif).

For each measurement, a recombinant GR ligand-binding domain (LBD) tagged with Glutathione-S-transferase (GST) was incubated for 30 min at room temperature with the motifs in Nuclear Receptor Buffer (Thermo Fisher Scientific, Waltham, MA, USA), together with a 50-fold diluted test sample or solvent control (2% DMSO). GR binding was detected using an ALEXA488-labeled anti-GST antibody, and unbound GR was removed by washing with assay buffer. Detection of bound GR receptor was performed by fluorescence microscopy and quantified using in-house R scripts (R Core Team, 2021). Cortisol (1 µM) served as a positive control for GC-induced GR activation.

## 3 Results

### 3.1 Identified Chinese search terms in CHHM-DB

Historical names for arthritis found in Chinese-language TCM journals may refer to both RA and PsA. None of the traditional names identified in the reviewed journal articles described the co-occurrence of arthritis and psoriatic skin lesions, and the names used for psoriasis did not appear in CHHM-DB.

Retrospective identification of biomedical disease entities in historical texts proved particularly challenging for dermatological conditions. Numerous historical disease names may represent psoriatic skin lesions. Conversely, a single historical term may refer to several different skin diseases with similar manifestations. E.g., the terms “purple patch wind” (*zidian feng*) and “bayberry sores” (*yangmei chuang*) are nowadays equated with lichen planus and syphilitic lesions, respectively. However, as diagnosis based on visual criteria alone remains unreliable (Gisondi et al., 2020; Schettini et al., 2021; Eskandari and Sharbatdar, 2024), it is possible that these terms were also used to describe certain manifestations of psoriasis. For arthritis, the traditional names used in TCM journals, such as “blockage condition” (*bizheng*) or “crane’s knee wind” (*hexi feng*), more closely match descriptions of arthritic conditions found in historical Chinese medical compendia. These terms also appear among the indications listed in CHHM-DB.

Through a comprehensive review of all indications in CHHM-DB, additional, more general terms such as “pain in the fingers” (*zhitong*), or “macules and papules” (*banzhen*) were identified as relevant search terms. Some frequently occurring indications, mostly skin-related, were excluded because historical descriptions suggested disorders unrelated to arthritis or psoriasis. E.g., the term “sores and ulcers” (*chuangyang*) refers to sores characterized by swelling, redness, and pus formation, thereby not corresponding to the most common manifestations of psoriasis.

The resulting list of 74 search terms defines the recipe dataset for PF analysis (see Supplementary Table 1 for the full list, including descriptions that reflect the similarities with clinical features of RA, PsA, and psoriasis). It includes:

1. 40 arthritis search terms of which 16 were clearly identifiable as arthritis (granularity “main” search terms) and 24 were less specific but related (granularity “orbit” search terms)
2. 34 skin-related search terms that resemble psoriatic lesions

**FIGURE 3:**
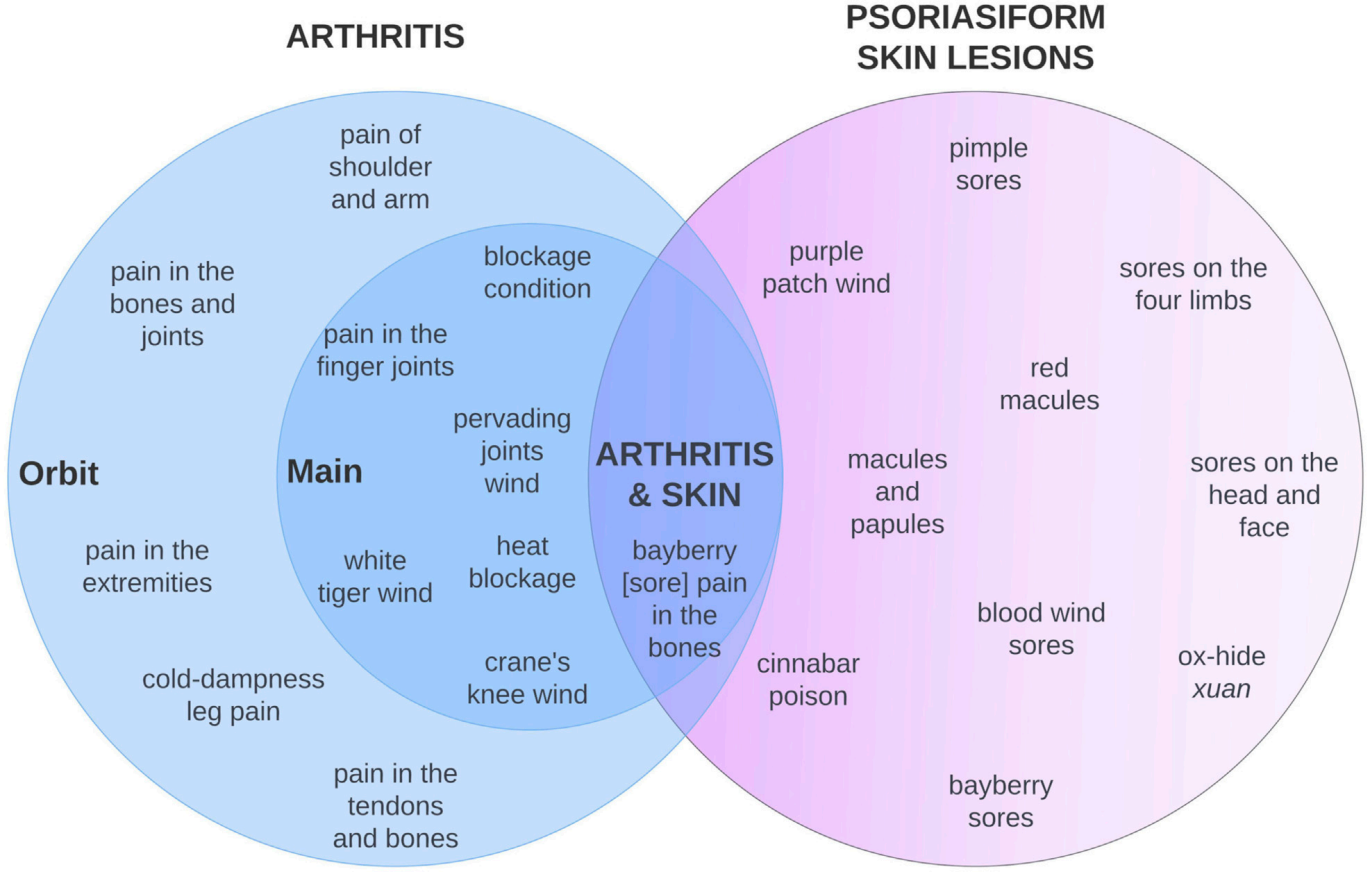
Venn diagram of exemplary search terms, differentiating between arthritis main and orbit indications (darker and lighter blue circles) and skin manifestations resembling psoriasis according to historical literature (those with a stronger resemblance are located further to the center). Search terms can consist of symptoms (e.g., “pain in the extremities”), disease names implying Chinese medicine pathologies (e.g., “blood wind sores”), or specific Chinese disease names (e.g., “white tiger wind”). The joint occurrence of arthritis and psoriasiform lesions may hint at the biomedical disease entity PsA.

Examples for the different groups of search terms are shown in Figure 3.

### 3.2 Data analysis

Based on the 74 search terms, an “All Search Terms” dataset consisting of 2,001 recipes and 682 botanical drugs was identified for data analysis. Of the ten most frequent search terms, four are related to skin conditions and six are related to arthritis indications (see Table 1). The four most frequent skin search terms cannot clearly be identified as psoriasis. They are linked to 746 recipes, constituting 67% of the category “skin”. Four of the six most frequent arthritis indications belong to granularity “orbit”, which designates musculoskeletal pain rather than joint inflammation. The six most frequent indications of the category “arthritis” are linked to 747 recipes, constituting 81% of all arthritis recipes (main and orbit). This indicates that a significant portion of the dataset is based on less precise search terms.

**TABLE 1:** Top 10 search terms according to their occurrence in recipes.

Top 10 search terms	Category	Granularity	Transliteration	Frequency
“Bayberry sores” (red sores on the body, usually equated with syphilitic sores)	Skin		*yangmei chuang*	474
“Pain of the tendons and bones”	Arthritis	Orbit	*jingu tong*	218
“Pain of lower back and legs”	Arthritis	Orbit	*yaotui tong*	183
“*gan*-illness sores” (various conditions of ulcers)	Skin		*ganchuang*	167
“leg pain”	Arthritis	Orbit	*tuitong*	155
“crane’s knee wind” (swelling and pain of the knee)	Arthritis	Main	*hexi feng*	78
“Wind blockage” (pain and stiffness of joints and muscles attributed to pathogenic “wind”)	Arthritis	Main	*feng bi*	57
“Bayberry knotted poison” (red sores on the body, usually equated with syphilitic sores)	Skin		*yangmei jiedu*	57
“Pain of bones and joints”	Arthritis	Orbit	*gujie tong*	56
“Macules and papules”	Skin		*banzhen*	52

The “All Search Terms” dataset contains a total of 216 botanical drugs that occur at least 10 times in these recipes. Implementing this threshold ensured the interpretability of the Pareto plots by excluding for example, obscure drugs with names that were too colloquial, dialectal, or ambiguous to be identified. Consequently, the dataset was reduced by one search term (*toumianxuan*) and 104 recipes, resulting in 73 search terms and 1,897 recipes. The resulting dimensions of the four subsets are as follows:

- Arthritis Main and Orbit: 899 recipes (275 Main + 624 Orbit)
- Arthritis Main: 275 recipes
- Skin: 1,032 recipes
- Arthritis&Skin: 34 recipes

Many of the 30 most frequent botanical drugs in the dataset, especially *Angelica sinensis* (Oliv.) Diels [Apiaceae] and *Glycyrrhiza* spp. [Fabaceae] (National Administration of TCM, 1999; National Pharmacopoeia Commission, 2020), were used widely in Chinese medicine to treat various inflammatory processes and/or pain, but also a wide range of other diseases (see Supplementary Data Sheet 1).

**FIGURE 4:**
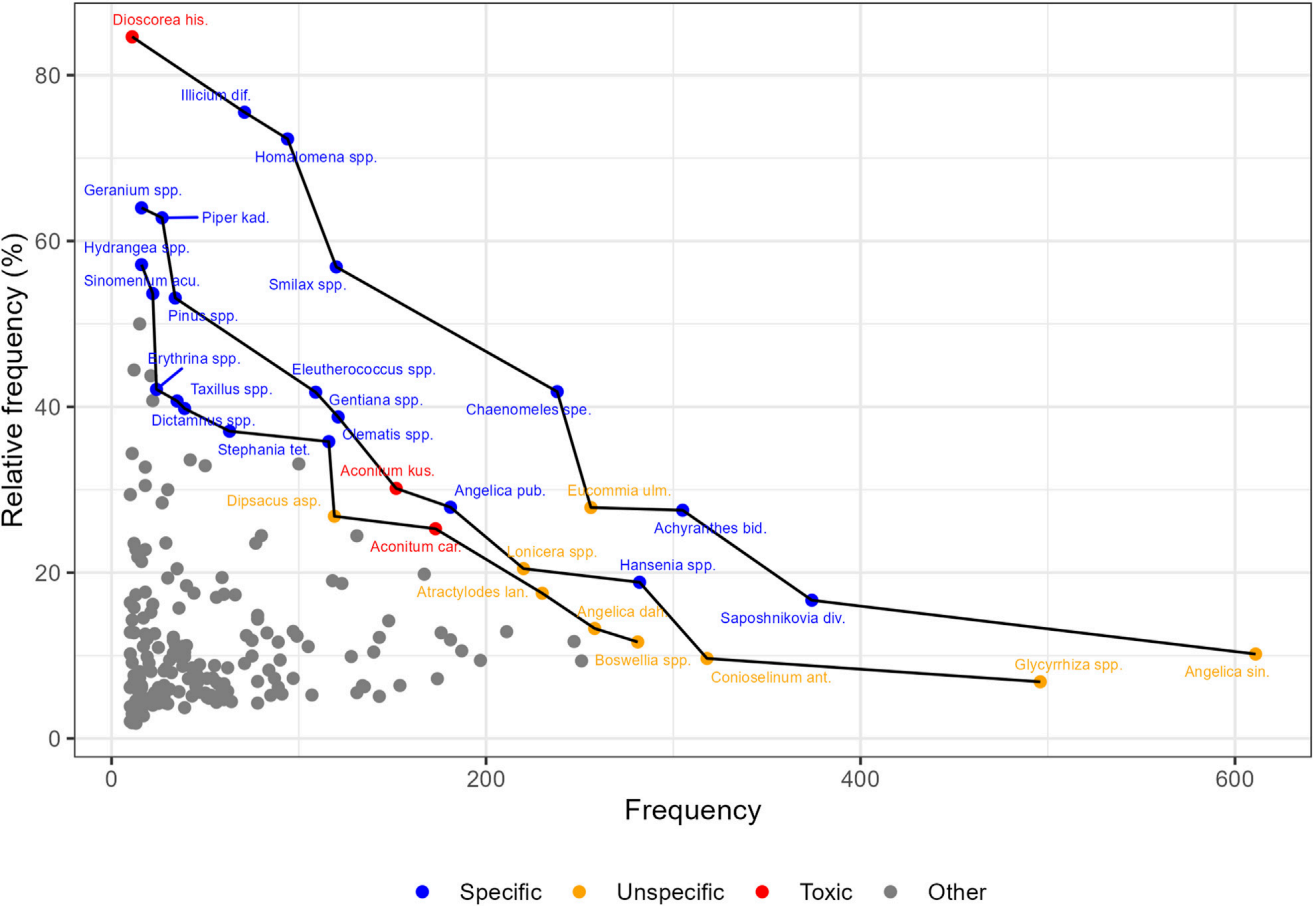
Pareto-optimal frequency of botanical drugs in recipes across all 73 search terms (“All Search Terms”). The x- and y-axes represent frequency and relative frequency of botanical drug occurrence, respectively. Black lines indicate Pareto front (PF) ranks 1–3, denoting the set of 32 candidates that show optimal combinations of both frequency metrics. Scientific names are abbreviated for readability (see Supplementary Table 2 for full taxonomic details). Colors represent classification according to Chinese *materia medica* (CMM): “specific” or “unspecific” usage for arthritis and/or psoriasiform skin lesions, or “toxic”. Botanical drugs below PF rank 3 (“Other”) were excluded from this exploratory analysis. The figure highlights that candidates with higher specificity for the targeted indications according to CMM coincide with higher relative frequency.

To determine which botanical drugs were used both frequently and specifically for the treatment of arthritis and psoriasiform skin diseases, we applied PF analysis to the “All Search Terms” dataset first. The outermost black line in Figure 4 constitutes PF rank 1. It connects the nine Pareto-optimal drugs in recipes linked to one or more of the 73 search terms. No other botanical drugs have a better balance of frequency and relative frequency in the “All Search Terms” dataset than these. Occurrences on PF rank 1 range from 11 occurrences in the “All Search Terms” dataset as opposed to 13 occurrences in the whole CHHM-DB dataset (i.e., relative frequency = 84.6%) of *Dioscorea hispida* Dennst. [Dioscoraceae] to 611 occurrences in the “All Search Terms” dataset as opposed to 6,000 occurrences in the whole CHHM-DB dataset (i.e., relative frequency = 10.2%) of *Angelica sinensis*. Similarly, 11 botanical drugs are distributed over rank 2, and 12 are distributed over rank 3. These 32 Pareto-optimal drugs appearing on ranks 1*–*3 constituted the list of candidates for screening. Frequency and relative frequency metrics of all Pareto-optimal candidates are listed in Supplementary Table 2.

**FIGURE 5:**
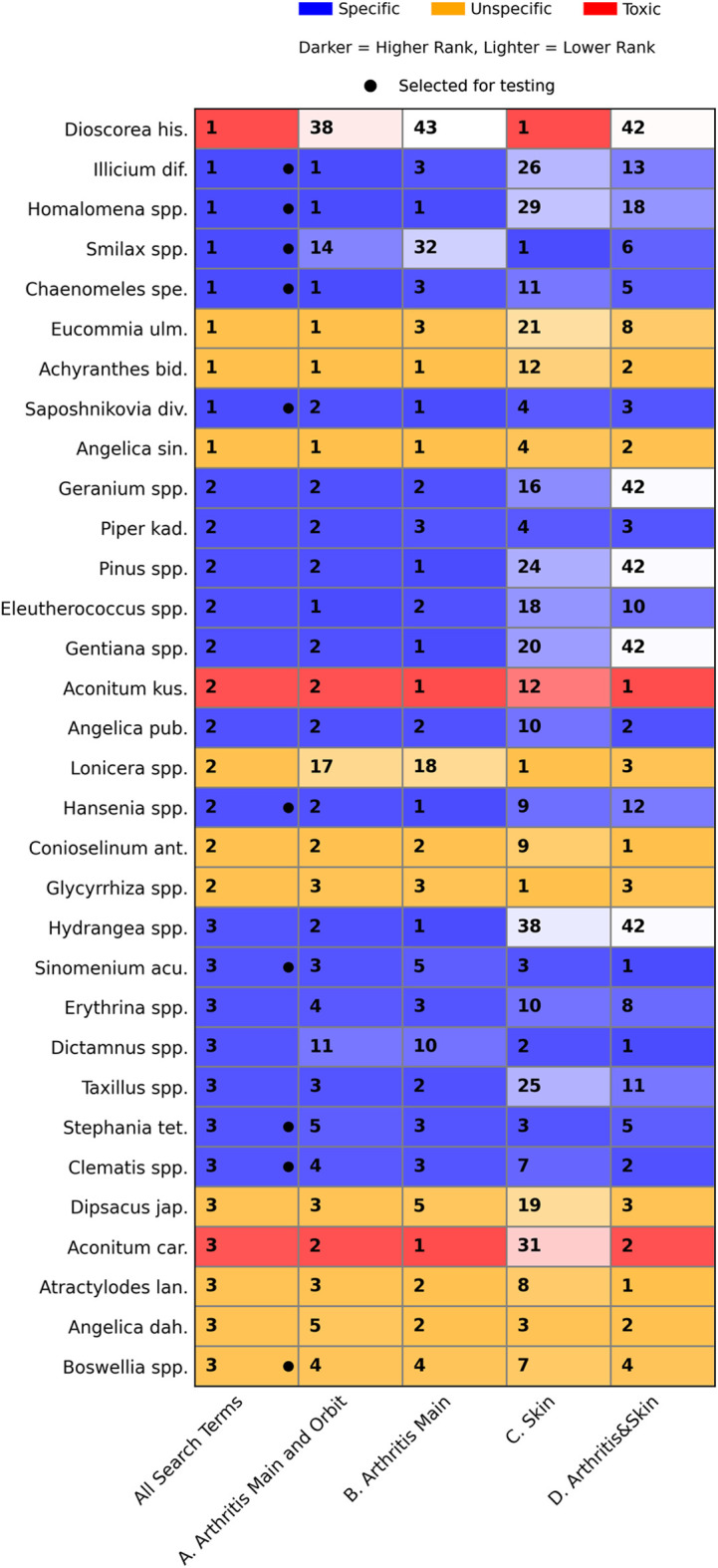
Heatmap showing Pareto-optimal candidates (rows) across search-term datasets (columns) derived from two broad groups of illnesses: arthritis (subdivided by indication specificity into A. “Main and Orbit” and B. “Main”) and psoriasiform skin lesions (“C. Skin”), as well as their intersection (“D. Arthritis and Skin”). Each cell indicates the Pareto Front (PF) rank of a given candidate within a specific dataset, with PF ranks designated by numbers. Candidates ranked 1–3 in the “All Search Terms” dataset are compared with their respective ranks in the subsets A–D. Colors denote candidate classification according to Chinese *materia medica* dictionaries: specific, unspecific, or toxic. Within each color category, darker shades represent higher (better) PF ranks, and lighter shades indicate lower ranks. Black dots mark candidates selected for screening. The figure illustrates how ranking of candidates varies across datasets defined by different search-term groups and how frequency and relative frequency in subsets A–D influence their presence on the PFs of the “All Search Terms” dataset.

In the next step, we explored how these results were influenced by the four subsets A*–*D by generating four separate PF analyses. Figure 5 presents the aggregated visualization of the results, combined with the findings from the CMM literature review (see also Supplementary Data Sheet 1, which displays the four plotted PFs for subsets A*–*D).

Among the 32 candidates appearing on PF ranks 1*–*3 of the “All Search Terms” dataset, 23 also ranked 1*–*3 in the arthritis subset A (“Arthritis Main and Orbit”), and 25 ranked 1*–*3 in subset B (“Arthritis Main”). In contrast, only eight botanical drugs ranking 1*–*3 in subset C (“Skin”) appear among the 32 candidates, while 15 ranking 1*–*3 in subset D (“Arthritis&Skin”) are found there. Comparing the arthritis subsets A and B with the skin subset C reveals that only three candidates (*Dioscorea hispida*, *Smilax* spp. [Smilacaceae], and *Lonicera spp*. [Caprifoliaceae]) stand out as being characteristic of the skin subset C, which explains their high ranking in “All Search Terms”.

### 3.3 Results of evaluation and comparison with contemporary TCM

All 32 candidates from the “All Search Terms” dataset were evaluated based on their monographs in CMM dictionaries, as described in the Methods section. Nineteen botanical drugs were classified as “specific”, 16 of which were traditionally recommended for arthritis, two (*Smilax* spp. and *Dictamnus* spp. [Rutaceae]) for psoriasiform skin lesions, and one (*Saposhnikovia divaricata* (Turcz.) Schischk. [Apiaceae]) for both. Ten botanical drugs were classified as “unspecific,” and three as “toxic”. As illustrated by the color distribution in Figure 4, plants with high relative frequency tended to be described in CMM as more specific (blue), whereas plants with high frequency but low relative frequency were often unspecific (orange).

Comparison of PF results with the *Chinese Pharmacopoeia* and PubMed showed that three plants (*Dioscorea hispida*, *Hydrangea* spp. [Hydrangeaceae] and *Erythrina* spp. [Fabaceae]) are no longer listed in the Chinese pharmacopoeia and have received little to no scientific attention. Of these, *Dioscorea hispida* and *Hydrangea* spp. have not been studied in the context of inflammatory diseases, while *Erythrina* spp. has only limited documentation, despite being described as specific in its CMM monographs.

None of the candidates still listed in the *Chinese Pharmacopoeia* have been studied in the context of PsA. Among those classified as “specific” or “toxic”, *Homalomena* spp. [Araceae], *Illicium difengpi* B. N. Chang [Schisandraceae] (PF rank 1), *Piper kadsura* (Choisy) Ohwi [Piperaceae], *Taxillus* spp. [Loranthaceae], and *Aconitum kusnezoffi* Rchb. [Ranunculaceae] (PF rank 2) have rarely been investigated for anti-inflammatory effects and even less research has focused on RA. In contrast, most other candidates have been studied in various *in vitro* and *in vivo* models. Unspecific but widely used botanical drugs such as *Glycyrrhiza* spp. and *Angelica sinensis* show the highest number of PubMed citations. Among the “specific” and “toxic” botanical drugs, *Sinomenium acutum* (Thunb.) Rehder & E.H.Wilson [Menispermaceae] stands out with 60 studies directly focused on RA, followed by *Smilax glabra* Roxb. [Smilacaceae], *Saposhnikovia divaricata*, and *Aconitum carmichaelii* Debeaux [Ranunculaceae], each with nearly 50 publications on their anti-inflammatory activity and several studies on RA and/or psoriasis. For all results from CMM evaluation and PubMed search metrics, see Supplementary Table 2.

Ten botanical drugs were selected for screening (see Table 2, cf. Figure 5 for their ranks in all subsets), including the five non-toxic, specific candidates from PF rank 1. Due to the exploratory nature of this study, only four specific candidates from PF rank 2 and 3 were selected. *Boswellia sacra* Flück. [Burseraceae], the only candidate described as unspecific in CMM, was included due to the widely known anti-inflammatory properties of different Boswellia species (Banno et al., 2006; Choudhary et al., 2023). The selected candidates are all listed in the *Chinese Pharmacopoeia* and were sourced at Shanghai pharmacies between September 2021 and September 2022.

**TABLE 2:** Chinese Materia Medica (CMM) evaluation results and PubMed search metrics of the ten plants selected for screening. PubMed search metrics include the number of articles on rheumatoid arthritis (RA), psoriatic arthritis (PsA), or psoriasis (Ps), as well as the number of articles on inflammation.

Latin name	Botanical family	Pharmaceutical name	Pareto front rank	CMM classi-fication	PubMed # RA, PsA, Ps	PubMed # inflam-mation
*Homalomena occulta* (Lour.) Schott	Araceae	Homalomenae Rhizoma	1	Specific	1 (RA)	2
*Illicium difengpi* B.N.Chang	Schisandraceae	Illicii Cortex	1	Specific	1 (RA)	5
*Smilax glabra* Roxb.	Smilacaceae	Smilacis glabrae Rhizoma	1	Specific	2 (RA), 6 (Ps)	48
*Chaenomeles speciosa* (Sweet) Nakai	Rosaceae	Chaenomelis Fructus	1	Specific	3 (RA)	20
*Saposhnikovia divaricata* (Turcz.) Schischk.	Apiaceae	Saposhnikoviae Radix	1	Specific	10 (RA), 1 (Ps)	48
*Hansenia weberbaueriana* (Fedde ex H.Wolff) Pimenov and Kljuykov [*Notopterygium incisum* Ting ex H. T. Chang]	Apiaceae	Notopterygii Rhizoma seu Radix	2	Specific	10 (RA)	31
*Sinomenium acutum* (Thunb.) Rehder and E.H.Wilson	Menispermaceae	Sinomenii Caulis	3	Specific	60 (RA), 1 (Ps)	92
*Stephania tetrandra* S.Moore	Menispermaceae	Stephaniae tetrandrae Radix	3	Specific	10 (RA), 2 (Ps)	31
*Clematis chinensis* Osbeck	Ranunculaceae	Clematidis Radix	3	Specific	14 (RA)	27
*Boswellia sacra* Flück.	Burseraceae	Olibanum	3	Unspecific	3 (RA)	36

### 3.4 Glucocorticoid receptor hit finding

In initial screening, the fractions extracted from the plant parts designated by the Chinese drug name were subjected to NAPing. GR-coregulator binding was measured in the presence of each plant fraction or cortisol (as control GC) and compared to that of unstimulated (Apo) GR.

**FIGURE 6:**
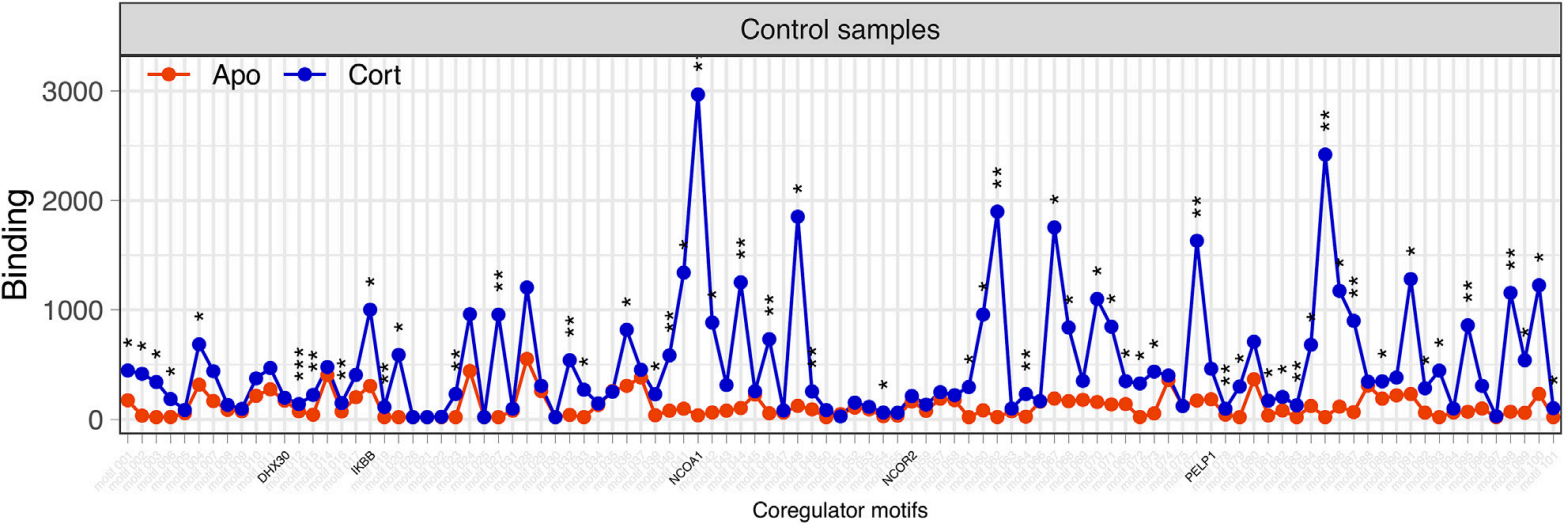
Binding profile of GR to 101 immobilized peptides of coregulator motifs under unstimulated and cortisol-stimulated conditions. Binding (y-axis) of unstimulated (Apo, red) or cortisol-stimulated (Cort, blue) GR to coregulator motifs (x-axis) was detected using a fluorescently labeled antibody and quantified. Both conditions were analysed using three technical replicates and are displayed as mean being ± S.E.M. The significance of cortisol-induced modulation (vs. Apo) of binding is shown as *p < 0.05; **p < 0.01; ***p < 0.001. The coregulator motifs representing either GC- or plant-induced activation (see Supplementary Table 3) that are later used for the analysis of plant fractions are highlighted in black. Cortisol evokes a robust and significant modulation of GR binding to a multitude of coregulator motifs including NCOA1.

As shown in Figure 6, cortisol significantly modulated the interaction of GR with multiple coregulator motifs. In particular, increased binding to NCOA1 (nuclear receptor coactivator 1 protein) (Liu et al., 1999) is considered the strongest identifying event for the presence of cortisol in canonical GR glucocorticoid-induced agonism (Zalachoras et al., 2013). NCOA1, therefore, was selected as a representative motif for cortisol-responsive GR-coregulator interactions (GC-motif).

During screening, plant fractions were analyzed as a single technical replicate, which did not permit statistical means for hit selection. Instead, potential hits were identified visually by plotting GR-coregulator binding across sequential fractions for each plant. Due to the fractionation procedure and the sequential screening of fractions in the series, fractions of interest are typically part of a continuous, bell-shaped activity gradient across adjacent fractions, allowing for visual identification. Such gradients were therefore interpreted as active fraction clusters containing potential GR-modulating plant metabolites.

None of the tested plant fractions induced the canonical cortisol-like binding pattern, i.e., modulation of GR interaction with the NCOA1 peptide was never observed. Instead, the active fraction clusters displayed alterations of GR binding to alternative coregulator motifs (plant-exclusive motifs).

**FIGURE 7:**
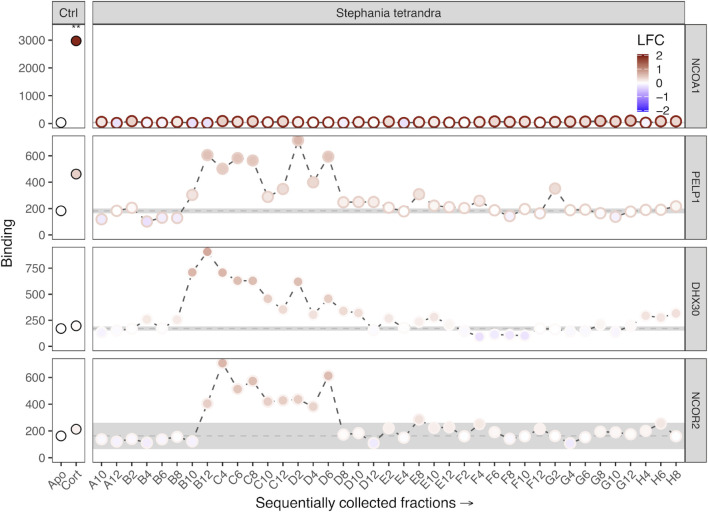
GR binding to the four selected GC- or plant-exclusive coregulator motifs (NCOA1, PELP1, DHX30, NCOR2). The left column of panels (Ctrl) shows GR interaction in the absence (Apo) or presence of cortisol (Cort) as the mean binding of three technical replicates. The significance of cortisol-induced modulation of binding is indicated as **p < 0.01. The right column of panels shows GR binding to the same motifs as modulated by sequentially collected plant fractions of *Stephania tetrandra*. The fill color of each data point indicates the sample-induced log-fold change (LFC) of GR-coregulator interaction relative to Apo binding. In the right panels, the edge color of data points represents cortisol-induced LFC of GR binding to the indicated coregulator motif. Apo GR binding (mean ± S.E.M.) is shown as a grey bar. GR-NCOA1 interaction is exclusively induced by cortisol. GR binding to the other represented coregulators is unaffected by this GC but is enhanced by all fractions of an active cluster (B12–D6) of *Stephania tetrandra*. These fractions do not affect GR-NCOA1 interaction.

A typical example of these findings is presented in Figure 7, which shows the effect of the fractions from *Stephania tetrandra* S. Moore [Menispermaceae] on GR, binding to the GC-motif (NCOA1) and three plant-exclusive motifs PELP1, DHX30, and NCOR2, with bell-shaped activity gradients across adjacent fractions. These motifs represent the coregulator proteins proline-, glutamic acid-, and leucine-rich protein 1 (Barletta et al., 2004), ATP-dependent RNA helicase DHX30 (Lessel et al., 2017), and nuclear receptor co-repressor 2 (Cui et al., 2011), respectively.

GR binding to NCOA1 was significantly enhanced over 1,000-fold by cortisol but not affected by any of the plant-derived fractions. Conversely, cortisol did not significantly change GR binding to PELP1, DHX30, or NCOR2, while an active cluster of sequential plant fractions displayed alterations of GR binding to these plant-exclusive motifs. To our knowledge, GC-induced GR interaction with either of these coregulators has never been reported.

In addition to mutually exclusive GR-coregulator interactions, we also found that GR-IKBB (NF-kappa-B inhibitor beta) (Liu et al., 2017) interaction is modulated by both cortisol and several tested plant fractions.

A total of four plants yielded five active fraction clusters (spanning a total of 34 individual fractions) that modulated GR-coregulator binding. All plants displayed a single activity cluster except for *Smilax glabra*, which showed two.

**FIGURE 8:**
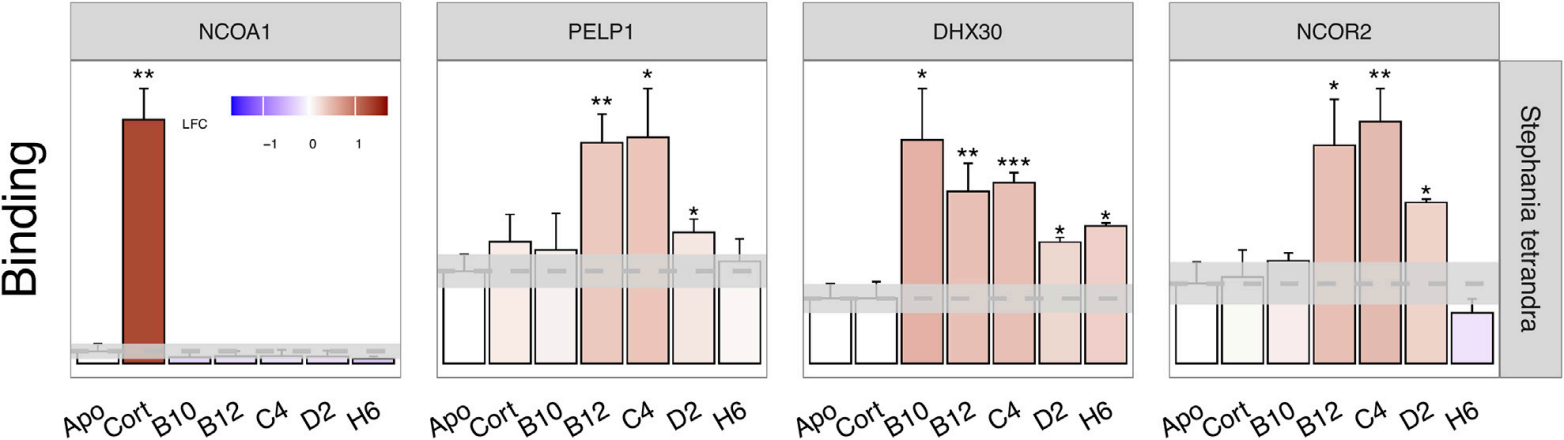
Statistical confirmation of the modulation of GR interaction with the four selected GC- or plant-exclusive coregulator motifs by cortisol (Cort) and a series of fractions from *Stephania tetrandra* (B10, B12, C4, D2, H6). Binding (y-axis), in arbitrary units’ fluorescence of the detection antibody, is shown as mean ± S.E.M. of three technical replicates. The significance of modulation of binding is shown as *p < 0.05; ** < 0.01; *** < 0.001. The bar color indicates the sample-induced log-fold change (LFC) of GR-coregulator interaction (vs. Apo). Apo GR binding level ± S.E.M. is indicated in the mean, dashed line (grey range). Only GR-NCOA1 interaction is exclusively and significantly induced by cortisol. GR binding to the other represented coregulators is unaffected by cortisol, but the gradual (significant) increase and decrease of binding as induced by an active fraction cluster of *Stephania tetrandra* follows the typical trend of a plant metabolites concentration gradient as a result of reverse phase separation and sample collection.

For confirmation, we selected a limited number of fractions that formed the apex of activity within each cluster. The presence of GR-modulating plant metabolites in each selected fraction was statistically confirmed by retesting in NAPing using three technical replicates. The results of this confirmation for *Stephania tetrandra* are shown in Figure 8.

### 3.5 Mechanism of action

**FIGURE 9:**
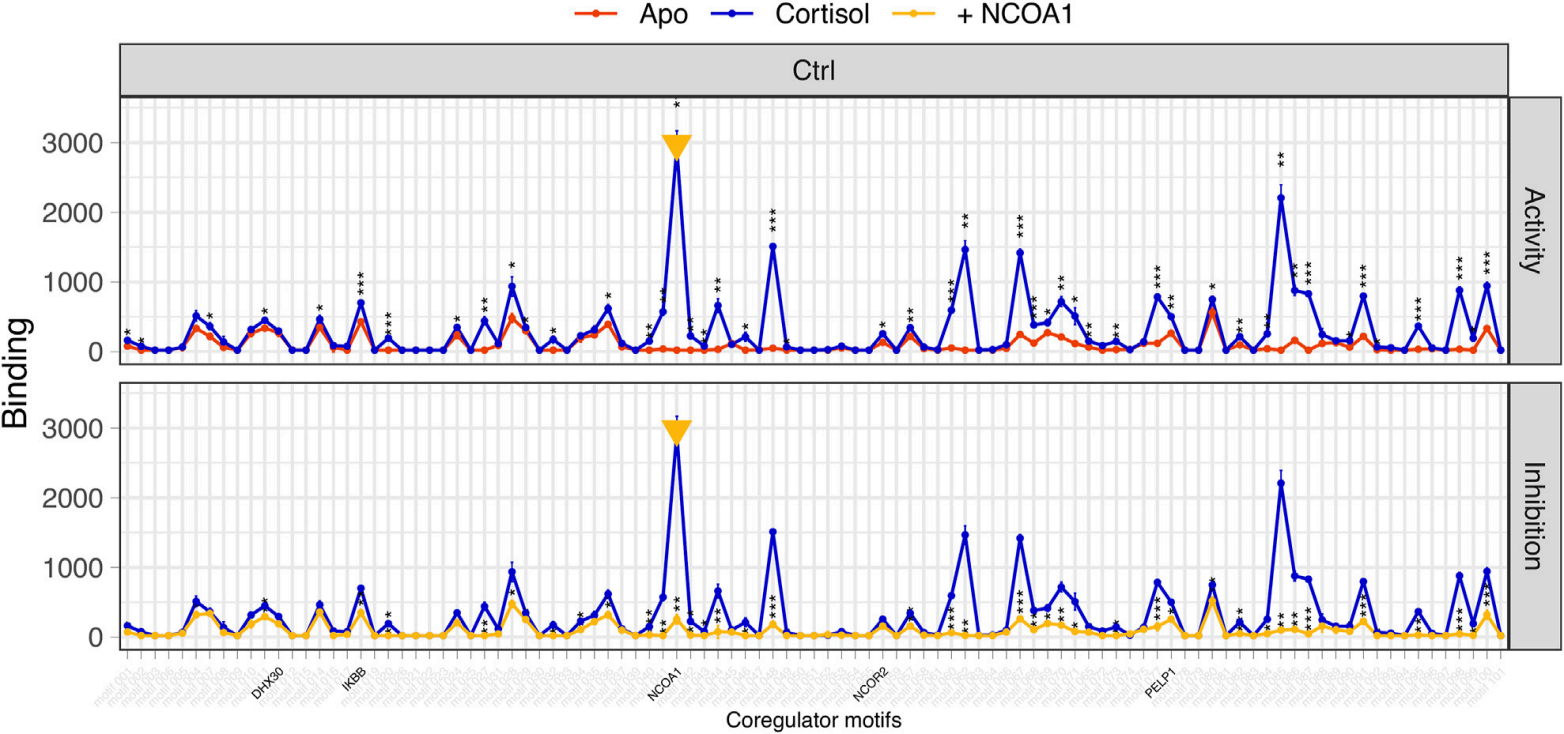
GC-dependent GR-coregulator interactions. GR binding (y-axis), in arbitrary units’ fluorescence of the detection antibody, is shown as mean ± S.E.M. of three technical replicates. Significance of GC-induced modulation and motif-induced inhibition of binding is indicated (*p < 0.05; ** < 0.01; *** < 0.001). GC- and plant-exclusive motifs are annotated in black. In the upper panel (activity), binding of unstimulated (Apo, red) GR to a multitude of coregulator motifs (including NCOA1, but not plant-exclusive motifs) is significantly enhanced upon GC stimulation (cortisol, blue). In the lower panel (inhibition), spiking the assay mix of cortisol-stimulated GR (blue) with NCOA1 coregulator motif (yellow arrow), competitively inhibited binding to all GC-responsive coregulators (yellow trace).

**FIGURE 10:**
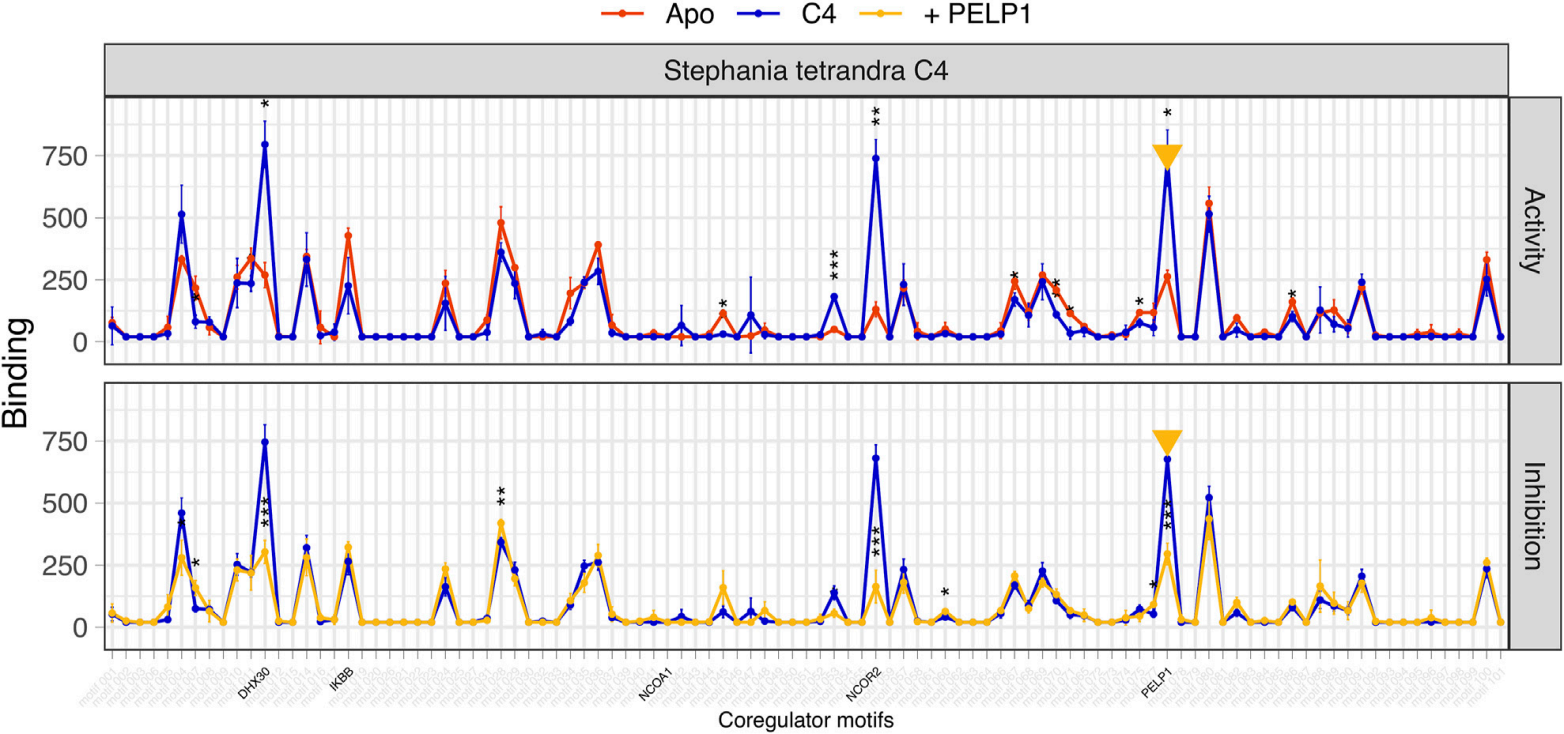
Plant-dependent GR-coregulator interactions. GR binding (y-axis), in arbitrary units’ fluorescence of the detection antibody, is shown as mean ± S.E.M. of three technical replicates. Significance of *Stephania tetrandra* C4-induced modulation and motif-induced inhibition of binding is indicated (*p < 0.05; ** < 0.01; *** < 0.001). GC- and plant-exclusive motifs are annotated in black. In the upper panel, binding of unstimulated (Apo, red) GR to a multitude of coregulator motifs (including highlighted plant-exclusive motifs, but not GC-exclusive motif NCOA1) is significantly enhanced upon stimulation (C4, blue). In the lower panel, spiking the assay mix of C4-stimulated GR (blue) with PELP1 coregulator motif (yellow arrow), competitively inhibited binding to all C4-responsive coregulators (yellow trace).

Based on the confirmation runs, the fraction with the highest activity within each of the five activity clusters was analyzed further to confirm the mechanism of action of GR-modulation. The GR protein is formed by 11-folded and interacting alpha-helices and a flexible helix-12. In the canonical way of GR coregulator-recruitment, GC binding stabilizes helix-12 of the receptor in such a way that a cleft is formed (GC-induced conformation), to which a coregulator motif can bind. Because all coregulator motifs interact with the same cleft in the GC-induced conformation in a one-to-one interaction, a strong-binding coregulator motif interferes with the binding of all others by competition. To illustrate this by NAPing, Figure 9 shows how cortisol-induced GR-coregulator interactions (upper panel) are all inhibited (lower panel) by spiking the solution with NCOA1 (the strongest binding motif to GR in GC-induced conformation). Because the tested plant fractions never appeared to induce GR-binding to NCOA1, we assume a possible alternative plant-induced GR conformation that facilitates recruitment and binding of alternative motifs. If this is the case, spiking of the assay solution with one of the plant-exclusive motifs should inhibit the interaction of GR in plant-induced conformation with all immobilized plant-exclusive motifs. To test this hypothesis, we selected PELP1, one of the plant-exclusive motifs recruited when GR is stimulated with fraction C4 of *Stephania tetrandra* (Figure 10, upper panel). When spiked to the assay solution, PELP1 significantly inhibits C4-stimulated GR binding to all plant-exclusive motifs (Figure 10, lower panel), suggesting that these all bind to the same (but distinct from GC-induced) site on GR in plant-induced conformation. In addition to *Stephania tetrandra* C4, a similar approach confirmed this mechanism for three out of the five selected fractions (see Table 3).

**TABLE 3:** Overview of the GR-modulating plant fractions identified by NAPing. Competitive inhibition of all NAPing interactions by addition of the indicated plant-exclusive motif in solution confirms an alternative GR conformation. “+” denotes confirmed inhibition, “*−*” non-confirmation (*Sinomenium acutum* C11), and “±” intermediate activity (*Smilax glabra* D1-D4).

Plant	Fraction position	Activity	Motif	Inhibition
*Saposhnikovia divaricata*	G10	+	IKBB	+
*Sinomenium acutum*	C11	−	IKBB	−
*Smilax glabra*	C5-C8	+	NCOR2	+
*Smilax glabra*	D1-D4	±	PELP1	±
*Stephania tetrandra*	C4	+	PELP1	+

## 4 Discussion

### 4.1 Short summary of findings

**FIGURE 11:**
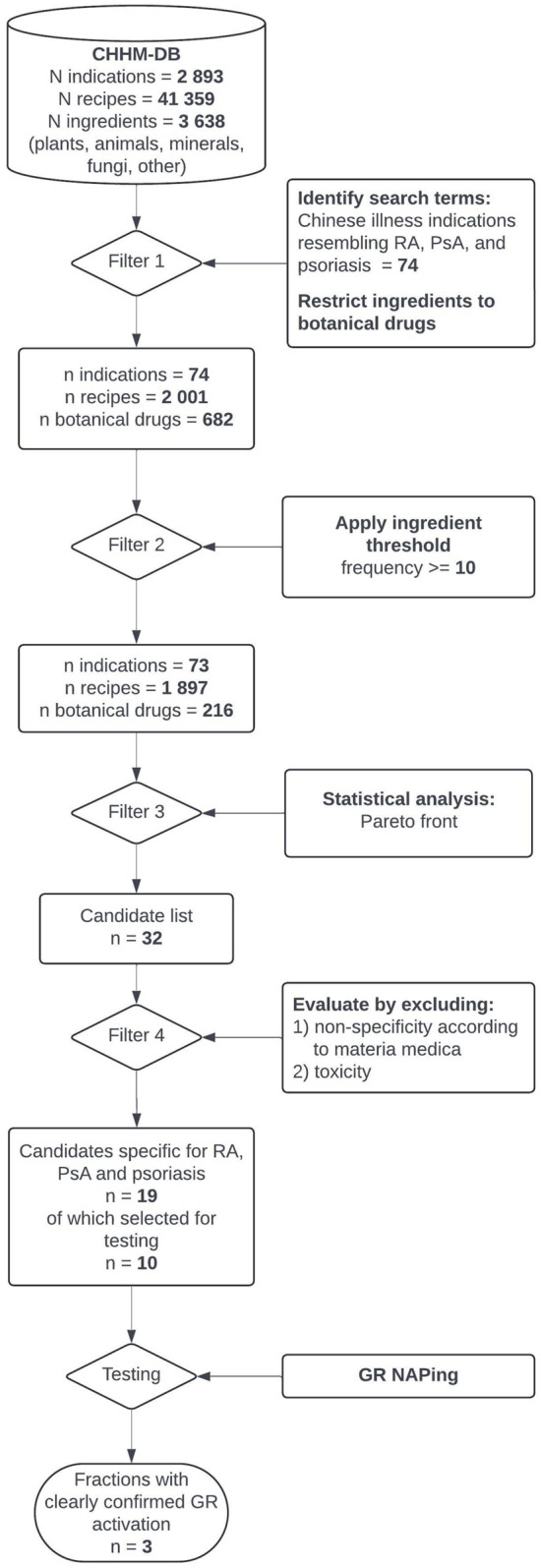
Multidisciplinary workflow depicting the steps required to select plants of interest to identify plant fractions that mediate GR activity.

This study combined historical, computational, and pharmacological approaches to identify botanical drugs with anti-inflammatory properties from historical CP recipes for the treatment of arthritis and psoriasiform skin diseases (see Figure 11 for a summary of the workflow). By integrating frequency and relative frequency through PF analysis on a dataset derived from CHHM-DB, 32 botanical drugs were identified. A literature review confirmed that 19 of these were historically described as specifically treating the targeted conditions. Extracts of 10 botanical drugs were fractionated and screened by NAPing to assess their effect on the GR, the pharmacological target of GC-based treatments for chronic inflammation. Thirty-four fractions showed modulation of GR activity. After confirmatory testing, five representative fractions were selected from four plants for further testing. While none reproduced canonical GC-mediated GR activity, three fractions from *Saposhnikovia divaricata, Smilax glabra,* and *Stephania tetrandra* modulated GR binding to alternative coregulator motifs, suggesting regulatory mechanisms distinct from classical GCs.

### 4.2 Insights on data mining historical sources

Mining historical Chinese medical texts for pharmacologically relevant botanical drugs presents distinct challenges and opportunities. The heterogeneity of disease nomenclature in premodern Chinese sources poses a general challenge to linking historical CP with biomedical disease entities (Barrett et al., 2015). In this study, the scarcity of detailed disease descriptions in the recipe manuscripts complicated the unambiguous identification of CHHM-DB indications as search terms. This limitation was particularly evident for psoriasis-related terminology, where, for example, *Dioscorea hispida* emerged as Pareto-optimal despite its main historical use for infectious skin diseases and injuries (National Administration of TCM, 1999; Nanjing University of TCM, 2014). Ambiguity proved advantageous, however, in the case of *Smilax glabra,* which appeared on PF rank 1 and was classified as “specific” through its specific association with disease names now generally interpreted as syphilitic lesions rather than psoriasis. Although rarely mentioned for the treatment of arthritis in either CMM or CHHM-DB, *Smilax glabra* has been reported to be effective not only against psoriasis (Guo et al., 2024) but also against RA (Dong et al., 2017). This usage spectrum, from syphilis to psoriasis to RA, suggests broad anti-inflammatory properties, which have already been well-described (Wu et al., 2022). The fact that fractions of *Smilax glabra* exhibited GR-modulating activity in the NAPing assay further adds to these insights.

The heatmap (Figure 5) illustrates how search term precision and the related size of subsets A*–*D influence Pareto optimization. Most candidates identified in the “All Search Terms” dataset also appear in ranks 1*–*3 of subset B (“Arthritis Main”), which, although comparatively small, is defined by precise search terms. In contrast, only three candidates are determined by the much larger subset C (“Skin”), which is based on more heterogeneous and less precise search terms. Consequently, “Arthritis Main” contains many botanical drugs associated specifically with arthritis, displaying well-balanced combinations of frequency and relative frequency, whereas “Skin” is dominated by a few highly specific botanical drugs alongside frequent but nonspecific ones. This suggests that varying terminology across disease categories in historical sources influences statistical results, emphasizing the importance of carefully defining search terms in text mining-based ethnopharmacological research.

PF analysis as a statistical method presents distinct advantages for data mining historical recipes. A comparison with the study by Xia et al. (2020), which also employed text mining in Chinese historical medical literature, highlights its added value. Xia et al. identify 31 botanical drugs for the treatment of RA in the printed texts of the *Encyclopedia of TCM* database. The majority of these plants are frequently used to treat a broad spectrum of diseases. In our dataset, as in Xia et al., frequency alone revealed commonly used botanical drugs but not the less common ones specifically associated with particular pathological conditions (see Supplementary Data Sheet 1). In contrast, as comparison with CMM shows, of the 32 botanical drugs identified through PF analysis in CHHM-DB, 16 are specifically recommended there for arthritis, two for psoriasiform skin lesions and one for both. Ten of the 16 arthritis-specific plants do not appear in Xia et al.’s results (see Supplementary Data Sheet 1). These differences confirm two key advantages of PF analysis: First, by considering not only frequency but also relative frequency, it reveals rare but highly specific botanical drugs such as *Sinomenium acutum, Hydrangea* spp. or *Erythrina* spp. Second, applying PF analysis to historical recipes using these inversely correlated metrics allows the detection of botanical drugs with moderate scores in both dimensions, such as *Smilax* spp. and *Stephania tetrandra*, which would remain unnoticed in single-metric analyses but emerge as Pareto-optimal due to their balanced metrics of interest.

Comparing the PF results derived from handwritten medical recipe records with the *Chinese Pharmacopoeia* and PubMed data revealed that a few specifically used botanical drugs from our dataset are less common in modern TCM, whereas most identified candidates remain well known. For the less common plants from PF rank 1 tested in the NAPing assay (*Homalomena occulta* and *Illicium difengpi*), no GR-modulating activity was observed. In contrast, three of their still widely used and well-researched counterparts (*Smilax glabra*, *Saposhnikovia divaricata,* and *Stephania tetrandra*) showed clear effects. The decline in use of once prominent botanical drugs may be due to multiple factors, and a negative result in a single screening method like NAPing does not exclude their pharmacological efficacy, especially when emerging studies suggest anti-inflammatory effects (Yang et al., 2019; Li et al., 2021). Further research is needed to assess whether the transmission of certain botanical drugs through the centuries correlates with their pharmacological activity, particularly for those that are historically significant but understudied.

### 4.3 Pharmacological implications

As Wainwright et al. note in their IUPHAR position review (Wainwright et al., 2022), drug discovery from natural sources is undergoing a renaissance driven by novel targets and advanced screening technologies. This highlights the pharmacological potential of plant metabolites in the search for new anti-inflammatory agents.

The currently available toolbox for lead identification in preclinical drug development offers a wide variety of cellular and *in vitro* assays. Several have uncovered bioactive plant metabolites with anti-inflammatory properties, including some acting through NR-related pathways (Hui et al., 2023; Wang et al., 2023). Because coregulator recruitment represents a critical early biological event following NR-ligand binding *in vivo*, we employed NAPing to assess the effects of isolated plant fractions on GR-coregulator binding.

Canonical activation of GR by GCs promotes the formation of a characteristic binding cleft that engages coregulators such as NCOA1 in a defined one-to-one interaction. In contrast, several plant-derived fractions in our study modulated GR-coregulator binding by showing distinct non-canonical coregulator preferences. Competition experiments confirmed that the observed plant-induced GR-coregulator binding also depends on a one-to-one interaction, which is distinct from that triggered by cortisol. Whether plant- and GC-induced complexes bind to the same position on the GR protein, each triggering a different conformation, or act *via* different interfaces remains to be investigated.

Coregulators constitute a diverse family of proteins with gene- and cell-specific functions, and their selective recruitment ultimately determines the transcriptional and pharmacological outcomes of ligand binding. Ligand-specific modulation of GR-coregulator recruitment therefore provides a mechanistic explanation for how subtle structural differences in ligands can translate into distinct biological responses. The discovery of such non-canonical GR modulation represents one of the most novel outcomes of this work. Although confirmation is needed, it may have uncovered a mechanism for selective GR modulation, in which plant-derived metabolites direct receptor activity towards alternative biological responses, thereby avoiding GC-related side effects. From a pharmacological perspective, this mechanism offers a promising direction towards future development of next-generation anti-inflammatory drugs.

While prior research on our final candidates *Sinomenium acutum* (Gong et al., 2023; Li et al., 2024), *Stephania tetrandra* (Song et al., 2024), *Saposhnikovia divaricata* (Jiang et al., 2024), and *Smilax glabra* (Ilyas et al., 2024) focused on well-characterized anti-inflammatory pathways, this study identifies an entirely different target profile, suggesting that underexplored plant fractions could yield metabolites acting as selective GR modulators with improved therapeutic windows.

### 4.4 Methodological limitations

When working with knowledge and data derived from historical manuscripts, several methodological constraints must be acknowledged. One important limitation is that it is not possible to conclusively identify the exact species used by the authors of the CHHM-DB manuscripts. When two or more botanical species are associated with the same Chinese name in modern CMM, the now typically used species was selected for screening, e.g., *Smilax glabra* Roxb. for *tufuling*, even though *Chinese Materia Medica* also lists the less common *Smilax laevis Wall. ex A.DC* [*Smilax lanceifolia* var. *opaca* A.DC., Smilacaceae] (National Administration of TCM, 1999). This choice may not represent historical usage in the manuscripts and may have influenced our screening results. Moreover, in historical literature as well as modern CMM dictionaries, discrepancies in naming occur due to the multitude of alternative plant names used in different geographic regions or historical periods. For example, although the Chinese name *fang ji* is equated with *Stephania tetrandra* in modern CMM, it may refer to other plants as well, including *Sinomenium acutum*, which the *Chinese Pharmacopoeia* lists under the Chinese name *qing feng teng* (Nanjing University of TCM, 2014; National Pharmacopoeia Commission, 2020). This study, like most modern research, relied on the standardized identification of plants, but local and historical naming habits remain an unresolved issue in ethnopharmacology.

CP has a long tradition of applying refined pre-preparation methods to raw drugs and of combining different plants within medical recipes to modify efficacy or toxicity. For instance, the anti-inflammatory effects of *Clematis chinensis* may be enhanced by thermal and wine processing (Jiang et al., 2022). Due to its exploratory stage, this study did not consider pre-prepared plants or plant combinations.

While PF analysis provided a multivariate approach superior to simple frequency counts, it remains a descriptive method based on non-controlled, historically biased samples, i.e., the distribution of indications and recipe ingredients in CHHM-DB is subject to factors like historical regional disease prevalence, availability of plants, and the personal therapeutic preferences of the manuscript authors. Although different statistical approaches allow for a normalization of bias such as varying number of recipes per indication, even logarithmic normalization remains descriptive. Despite these limitations, PF analysis nevertheless yielded meaningful candidates, as supported by their classification in CMM and PubMed search results, confirming that most of these plants are known for their anti-inflammatory properties.

### 4.5 Future research

Future data analyses could build upon the current PF-based approach by incorporating advanced machine-learning techniques, including retrieval-augmented generation, to better contextualize CHHM-DB data within larger corpora of digitized historical medical texts, such as *Drugsacrossasia_China* (Stanley-Baker et al., 2023). These methods may enable the detection of more complex patterns and associations between recipes, plant usage, and the heterogeneous therapeutic concepts present in historical Chinese medicine.

Building on the promising NAPing results, subsequent studies should aim to identify and purify the bioactive metabolites within the plant fractions that are responsible for the observed GR-modulating activity. NAPing could initially guide these efforts to monitor activity and the mechanism of action. The pharmacological profiles of these purified plant metabolites should then be compared with GCs in preclinical *in vitro* and *in vivo* inflammation models. The differential recruitment of coregulators, as suggested by NAPing, and our proposed alternative mechanism of action require confirmation through orthogonal technologies.

More broadly, the combined use of historical pharmacology, data mining, and *in vitro* screening approaches such as NAPing, offers a transferable framework that can be applied to other disease categories and traditional medical systems. Such integrative strategies may continue to uncover overlooked effects of medicinal plants and their biologically relevant metabolites, as well as, illuminate novel mechanisms for target engagement.

## 5 Conclusion

This study demonstrates the potential of reverse-translational research that bridges historical pharmaceutical practice with modern molecular pharmacology. By combining data mining of historical Chinese medical recipes with an NR assay, we identified plant-derived fractions that modulate GR through non-canonical interaction patterns. Future work is needed to isolate and characterize the active metabolites responsible for these effects and to validate their selective modulation of GR in complementary experimental systems. Such efforts may ultimately contribute to the development of more targeted and possibly safer GR-based therapies for chronic inflammatory diseases. Overall, our findings highlight the potential of integrating ethnopharmacological knowledge with *in vitro* screening to open new avenues for drug discovery.

## Data Availability

The raw data supporting the conclusion of this article will be made available by the authors, without undue reservation.
