# Supplementary material for: Data mining historical Chinese medical recipe collections and nuclear receptor profiling identify plant fractions that modulate glucocorticoid receptor activity
Source: Front Pharmacol. 2026 Jan 7;16:1681729. doi: 10.3389/fphar.2025.1681729 (PMC12819651; doi:10.3389/fphar.2025.1681729)

Supplementary Material - Prackwieser et al. **Data Mining Historical Chinese Medical Recipe Collections and Nuclear Receptor Profiling Identify Plant Fractions that Modulate Glucocorticoid Receptor Activity.**

Table 1. Top 30 botanical drugs out of the 216 occurring more than 10 times in the "All Search Terms" dataset.

#	Scientific name	Family	Pharmaceutical name	Transliteration	Frequency ("All Search Terms" dataset)	Frequency (CHHM-DB)	Relative Frequency (%)
1.	*Angelica sinensis* (Oliv.) Diels	Apiaceae	Angelicae sinensis Radix	*danggui**	611	6000	10.2
2.	*Glycyrrhiza inflata* Batalin; *Glycyrrhiza uralensis* Fisch. ex DC.; *Glycyrrhiza glabra* L.	Fabaceae	Glycyrrhicae Radix	*gancao**	496	7257	6.8
3.	*Saposhnikovia divaricata* (Turcz. ex Ledeb.) Schischk.	Apiaceae	Saposhnikoviae Radix	*fangfeng**	374	2243	16.7
4.	*Conioselinum anthriscoides* (H.Boissieu) Pimenov & Kljuykov [*Ligusticum chuanxiong* S.H.Qiu, Y.Q.Zeng, K.Y.Pan, Y.C.Tang & J.M.Xu]	Apiaceae	Chuanxiong Rhizoma	*chuanxiong**	318	3294	9.7
5.	*Achyranthes bidentata* Blume	Amaranthaceae	Achyranthis bidentatae Radix	*niuxi**	305	1108	27.5
6.	*Hansenia weberbaueriana* (Fedde ex H.Wolff) Pimenov & Kljuykov *[Notopterygium incisum* Ting ex H. T. Chang]; *Hansenia forbesii* (H.Boissieu) Pimenov & Kljuykov *[Notopterygium forbesii* H.Boissieu]	Apiaceae	Notopterygii Radix et Rhizoma	*qianghuo**	282	1497	18.8
7.	*Boswellia sacra* Flück. [*Boswellia carteri* Birdw.]; *Boswellia neglecta* S.Moore	Burseraceae	Olibanum	*ruxiang**	281	2414	11.6
8.	*Angelica dahurica* (Hoffm.) Benth. & Hook.f. ex Franch. & Sav.	Apiaceae	Angelicae Dahuricae Radix	*baizhi**	258	1944	13.3
9.	*Eucommia ulmoides* Oliv.	Eucommiaceae	Eucommiae Cortex	*duzhong**	256	919	27.9
10.	*Rheum palmatum* L.; *Rheum officinale* Baill.; *Rheum tanguticum (Maxim. ex Regel) Balf.*	Polygonaceae	Rhei Radix et Rhizoma	*dahuang*	251	2683	9.4
11.	*Commiphora myrrha* (T.Nees) Engl.	Burseraceae	Myrrha	*moyao*	247	2112	11.7
12.	*Chaenomeles speciosa* (Sweet) Nakai	Rosaceae	Chaenomelis Fructus	*mugua*	238	569	41.8
13.	*Atractylodes lancea* (Thunb.) DC.	Asteraceae	Atractylodis Rhizoma	*cangzhu**	230	1313	17.5

14.	*Lonicera japonica* Thunb.; *Lonicera hypoglauca* Miq.; *Lonicera confusa* DC.; *Lonicera macrantha* (D.Don) Spreng.	Caprifoliaceae	Lonicerae Flos	*jinyinhua*	220	1074	20.5
15.	*Neolitsea cassia* (L.) Kosterm. [*Cinnamomum cassia* (L.) J.Presl]	Lauraceae	Cinnamomi Ramulus	*rougui**	211	1638	12.9
16.	*Rehmannia glutinosa (Gaertn.)* Libosch. ex DC.	Orobanchaceae	Rehmanniae Radix	*shengdihuang**	197	2092	9.4
17.	*Rehmannia glutinosa (Gaertn.)* Libosch. ex DC.	Orobanchaceae	Rehmanniae Radix preparata	*shudihuang**	187	1770	10.6
18.	*Angelica pubescens* Maxim.*; Angelica biserrata* (R.H.Shan & C.Q.Yuan) C.Q.Yuan & R.H.Shan	Apiaceae	Angelicae pubescentis Radix	*duhuo*	181	649	27.9
19.	*Phellodendron amurense* Rupr.; *Phellodendron chinense* C.K.Schneid.	Rutaceae	Phellodendri Cortex	*huangbai**	181	1521	11.9
20.	*Nepeta tenuifolia* Benth. [*Schizonepeta tenuifolia* (Benth.) Briq.]	Lamiaceae	Schizonepetae Herba	*jingjie*	176	1380	12.8
21.	*Dryobalanops aromatica* C.F.Gaertn.; *Blumea balsamifera* (L.) DC.	Dipterocarpaceae; Asteraceae	Borneolum	*bingpian*	174	2415	7.2
22.	*Aconitum carmichaelii* Debeaux	Ranunculaceae	Radix Aconiti	*chuanwu**	173	684	25.3
23.	*Ephedra sinica* Stapf; *Ephedra intermedia* Schrenk & C.A.Mey.; *Ephedra equisetina* Bunge	Ephedraceae	Ephedrae Herba	*mahuang**	167	843	19.8
24.	*Atractylodes macrocephala* Koidz.	Asteraceae	Atractylodis macrocephalae Rhizoma	*baizhu**	154	2410	6.4
25.	*Aconitum kusnezoffii* Rchb.	Ranunculaceae	Aconiti kusnezoffii Radix	*caowu**	152	504	30.2
26.	*Forsythia suspensa* (Thunb.) Vahl	Oleaceae	Forsythiae Fructus	*lianqiao*	148	1043	14.2
27.	*Citrus reticulata* Blanco	Rutaceae	Citri Reticulatae Pericarpium	*chen pi**	143	2814	5.1
28.	*Paeonia lactiflora* Pall.; *Paeonia veitchii* Lynch; *Paeonia obovata* Maxim.; *Paeonia obovata* subsp. *willmottiae* (Stapf) D.Y.Hong & K.Y.Pan; *Paeonia mairei* H.Lév.; *Paeonia anomala* L.; *Paeonia intermedia* C.A.Mey.	Paeoniaceae	Paeoniae rubra Radix	*chishao**	143	1173	12.2
29.	*Carthamus tinctorius* L.	Asteraceae	Carthami Flos	*honghua*	140	1343	10.4
30.	*Scutellaria baicalensis* Georgi; *Scutellaria amoena* C.H.Wright; *Scutellaria viscidula* Bunge; *Scutellaria likiangensis* Diels	Lamiaceae	Scutellariae Radix	*huangqin**	135	2162	6.2

* These botanical drugs appear also in the results of Xia *et al.* (2020)

Figure 1. Sample recipe from the Unschuld Collection. Manuscript ID 8282 (“Secret Recipes Handed Down from Ancestors” 祖传秘方), p. 109 (CHHM-DB pos. 257/312). This recipe goes back to the book “Correcting Errors Among Physicians” by Wang Qingren 王清任 (1768–1831), but the entry in the manuscript omits Wang’s theoretical elaborations on the pathophysiological causes of pain. Link to scan: https://digital.staatsbibliothek-berlin.de/werkansicht/?PPN=PPN3346231216&PHYSID=PHYS_0109. Owning institution: Stabi Berlin.

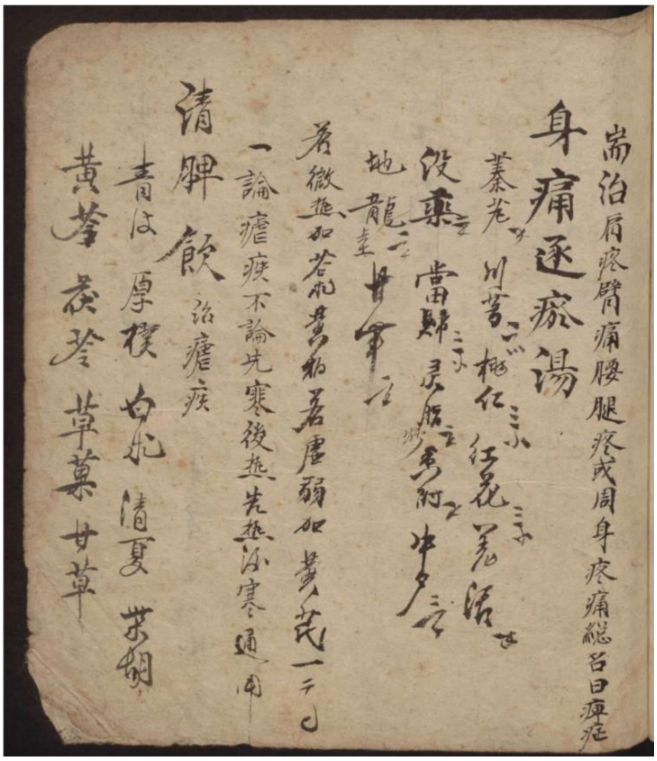

Transcription of Manuscript ID 8282 祖传秘方, p. 109 (CHHM-DB pos. 257/312):

专治肩疼臂痛，腰腿疼，或周身疼痛，总名曰痹症。

[Indications:] “Specifically treats pain in the shoulders and arms, pain in the lower back and legs, or pain in the whole body. This is generally called blockage condition.”

身痛逐瘀汤

[Recipe name:] “Decoction that drives out stasis from a painful body”

秦艽一钱 川芎二钱 桃仁三钱 红花三钱 羌活一钱 没药二钱 当归三钱 灵脂二钱 香附一钱 牛夕三钱 地龙二钱去土 甘草一钱

[Ingredients and dosage:] “Large Leaf Gentian root – 1 *qian*, Sichuan Lovage rhizome – 2 *qian*, Peach kernels – 3 *qian*, Safflower – 3 *qian*, Notopterygium root – 1 *qian*, Myrrh – 2 *qian*, Chinese Angelica root – 3 *qian*, Flying Squirrel feces – 2 *qian*, Nut-grass rhizome – 1 *qian*, Oxknee root – 3 *qian*, Earthworm – 2 *qian* (remove the earth), Sweetwood – 1 *qian*.”

若微热，加 苍术 黄柏。若虚弱，加 黄芪一、二两。

[Modifications:] "In case of a little heat, add Atractylodes root and Phellodendron bark. In case of depletion and weakness, add one or two *liang* of Astragalus root." [1]

[1] *Qian* (mace): historical weight unit, approx. 3.7 g in Late Imperial China. Ten *qian* make one *liang* (tael). We use historical unit names and the common names of recipe ingredients in the translation to retain the historical character of the text.

Figure 2. Pareto-optimal plants on the top 3 Ranks of each of the four subsets: A. "Arthritis Main and Orbit" recipes subset, B. "Arthritis Main subset", C. "Skin" subset, and D. "Arthritis&Skin" subset that includes recipes indicated for arthritis as well as psoriasiform skin lesions. Plants that do not appear in the "All Search Terms" dataset are greyed out. The doubling of *Cinnamonum cassia* in A. and *Glycyrrhiza* spp. in C. is due to the differentiation of plant parts in the dataset.

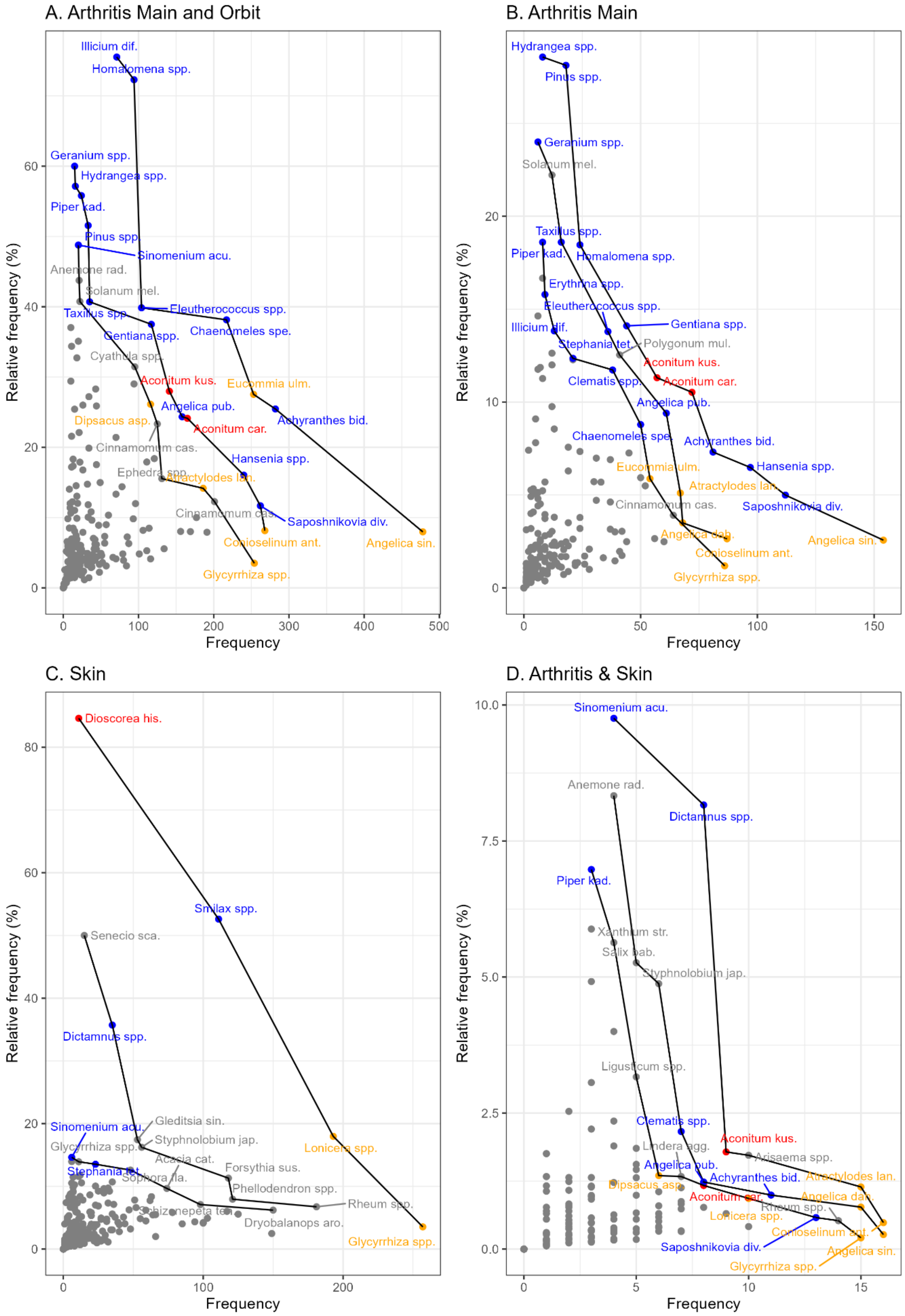

Table 2. Botanical drugs on PF 1-3 in subset A-D, which do not appear on PF 1-3 in "All Search Terms".

Scientific name	Family	Pharmaceutical name	Transliteration	On PF 1-3 in subset
Senegalia catechu (L.f.) P.J.H.Hurter & Mabb. [*Acacia catechu* (L.f.) Willd.]*	Fabaceae	Catechu	*ercha*	C
Anemone raddeana Regel	Ranunculaceae	Anemones Raddeanae Rhizoma	*liangtoujian*	A,D
Arisaema amurense Maxim.; *Arisaema erubescens* (Wall.) Schott; *Arisaema heterophyllum* Blume	Araceae	Arisaematis Rhizoma	*tiannanxing*	D
Neolitsea cassia (L.) Kosterm. [*Cinnamomum cassia* (L.) J.Presl]*	Lauraceae	Cinnamomi Ramulus	*rougui*	A,B
Neolitsea cassia (L.) Kosterm. [*Cinnamomum cassia* (L.) J.Presl]*	Lauraceae	Cinnamomi Ramulus	*guizhi*	A
Cyathula officinalis K.C.Kuan	Amaranthaceae	Cyathulae Radix	*chuanniuxi*	A
Dryobalanops aromatica C.F.Gaertn.; *Blumea balsamifera* (L.) DC.	Dipterocarpaceae; Asteraceae	Borneolum	*bingpian*	C
Ephedra sinica Stapf; *Ephedra intermedia* Schrenk & C.A.Mey.; *Ephedra equisetina* Bunge	Ephedraceae	Ephedrae Herba	*mahuang**	A
Forsythia suspensa (Thunb.) Vahl	Oleaceae	Forsythiae Fructus	*lianqiao*	C
Gleditsia sinensis Lam.	Fabaceae	Gleditsiae abnormalis Fructus	*zaojiao*	C
Conioselinum anthriscoides (H.Boissieu) Pimenov & Kljuykov [*Ligusticum sinense* Oliv.]; *Conioselinum smithii* (H.Wolff) Pimenov & Kljuykov [*Ligusticum jeholense* (Nakai & Kitag.) Nakai & Kitag.]*	Apiaceae	Ligustici Rhizoma et Radix	*gaoben*	D
Lindera aggregata (Sims) Kosterm.	Lauraceae	Linderae Radix	*wuyao*	D
Phellodendron amurense Rupr.; *Phellodendron chinense* C.K.Schneid.	Rutaceae	Phellodendri Cortex	*huangbai**	C
Reynoutria multiflora (Thunb.) Moldenke [*Polygonum multiflorum* Thunb.]	Polygonaceae	Polygoni multiflori Radix	*heshouwu*	B
Rheum palmatum L.; *Rheum officinale* Baill.; *Rheum tanguticum* Maxim. ex Balf.	Polygonaceae	Rhei Radix Et Rhizoma	*dahuang*	C,D
Salix babylonica L.	Salicaceae	Salicis babylonicae Ramulus	*liuzhi*	D
Nepeta tenuifolia Benth. [*Schizonepeta tenuifolia* (Benth.) Briq.]*	Lamiaceae	Schizonepetae Herba	*jingjie*	C
Senecio scandens Buch.-Ham. ex D.Don	Asteraceae	Senecionis Scandentis Herba	*qianliguang*	C
Solanum melongena L.	Solanaceae	Solani Melongenae Radix	*qiegen*	A,B

Sophora flavescens Aiton	Fabaceae	Sophorae flavescentis Radix	*kushen*	C
Styphnolobium japonicum (L.) Schott	Fabaceae	Sophorae Flos	*huaihua*	C,D
Xanthium strumarium L.	Asteraceae	Fructus Xanthii	*cangerzi*	D

* In these cases, abbreviated labels in subplots A-D reflect the commonly used botanical drug names, as highlighted in bold.

Figure 3. Venn diagram comparing Xia *et al.* (2020) results with PF analysis results of CHHM-DB. Fifteen botanical drugs were identified by both approaches, 17 only by PF and 14 only by Xia et al., the latter all constituting botanial drugs widely used in Chinese medicine. Sixteen arthritis-specific botanical drugs were produced by PF (blue), out of which ten appear only in CHHM-DB/PF results. Two botanical drugs from PF are specific for psoriasiform skin lesions (pink), one for both arthritis and skin lesions (purple), nine are unspecific (orange) and three toxic (red).

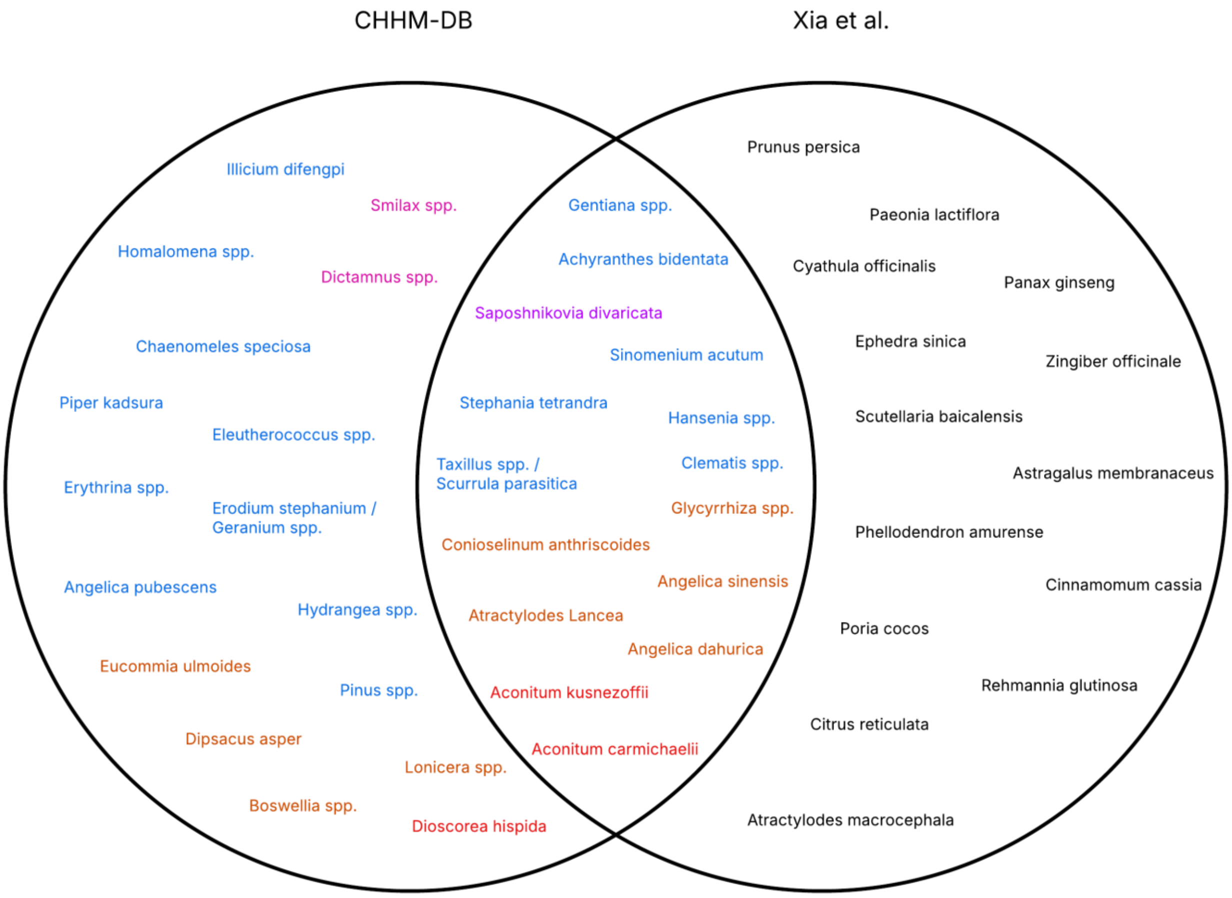

Supplement: Supplementary file 3 [file DataSheet1.pdf]
